# Convective modes reveal the incoherence of the Southern Polar Vortex

**DOI:** 10.1038/s41598-023-50411-x

**Published:** 2024-01-10

**Authors:** Chantelle Blachut, Sanjeeva Balasuriya

**Affiliations:** School of Computer and Mathematical Sciences, University of Adelaide, Adelaide, SA 5005 Australia

**Keywords:** Climate sciences, Atmospheric science, Atmospheric dynamics, Mathematics and computing, Applied mathematics, Atmospheric dynamics

## Abstract

The Southern Polar Vortex (SPV) is prominent over Antarctica in the Austral winter, and typically associated with a region of low temperature, low ozone concentration, negative potential vorticity, and polar stratospheric clouds. Seasonal and unexpected changes in the SPV have a profound influence on global weather. A methodology which identifies the SPV’s coherence and breakup using only wind and pressure data is developed and validated against temperature, ozone and potential vorticity data. The process identifies “convective modes”, each with an assigned “coherence” value, which form building blocks for the observed spatial variation of the SPV. Analysis and interpretation are presented for 4 years with quite different known behavior of the SPV: 1999 (a relatively standard year), 2002 (when the SPV split into two), 2019 (an atmospheric warming year which led to an early dissipation in the SPV), and the most recent year 2022 (which was influenced by submarine volcano eruptions and a prolonged La Niña event). In decomposing convective effects into modes with quantifiable coherence, this study solidifies connections between wind velocities and atmospheric variables while providing new tools to study the evolution of coherent structures and signal the occurrence of atypical geophysical events.

## Introduction

Each year, a large mass of cold air gathers in the stratosphere above Antarctica and heralds the austral winter. This mass, more commonly known as the Southern Polar Vortex (SPV), is contained by a strong jet stream that allows it to evolve high above the polar regions. While it remains coherent, the SPV is further characterized by strong clockwise (cyclonic) rotation, potential vorticity anomalies, large volumes of polar stratospheric clouds, and low concentrations of ozone. Sometime between September and early October each year, the SPV experiences translation and eventual decay^1^. Prevailing wind patterns clearly influence the coherence and dissipation of the SPV, which in turn impacts the spatio-temporal distribution of atmospheric variables. Our research exploits this relationship to effectively characterize when and how rare events, such as sudden stratospheric warmings (SSW), impact the evolution of the SPV and consequently weather conditions at Earth’s surface^2^.

While the SPV’s coherence and decay follow a similar trend and spatial distribution each year, unexpected variations from this timeline can dramatically impact weather patterns. We study 4 years in which the SPV evolved in different ways, 1999, 2002, 2019 and 2022. In 1999, an unremarkable Southern Oscillation Index (SOI) throughout the Winter months coincided with an SPV whose initial behavior was fairly typical^3–5^, despite blocking activity (stationary regions of exceptional pressure) centered around New Zealand and more mobile and shorter-lived blocks elsewhere^6^. Late 2002 saw a dramatic event in which the single SPV split into two vortices. This anomaly was associated with a major stratospheric warming event, and has been analyzed in detail^1,7,8^. A slower warming event accompanied the Australian wildfire season of late 2019^2,9–13^. Finally, 2022, the most recent year for which data is available, was characterized by the remarkable eruption of Hunga Tonga-Hunga Ha’apai which sent aerosols deep into the stratosphere and the conclusion of a prolonged predominance of positive SOI values.

We investigate the SPV at a stratospheric level and develop the concept of “convective modes” derived solely from wind conditions over the previous fortnight. Each mode is associated with a “coherence” value that identifies its relative importance. Importantly, these modes are spatially extended structures that prove to be excellent predictors of the distribution of atmospheric variables. Moreover, we track their movement and changes in coherence over time to show how spatial variations, dictated by rare events, become established and well-defined structures fall in prominence as the SPV undergoes decay. This methodology provides an excellent tool for the analysis of interactions between anomalous events and the SPV, e.g., when SSW or other atypical events disturb regular seasonality.

**Figure 1:**
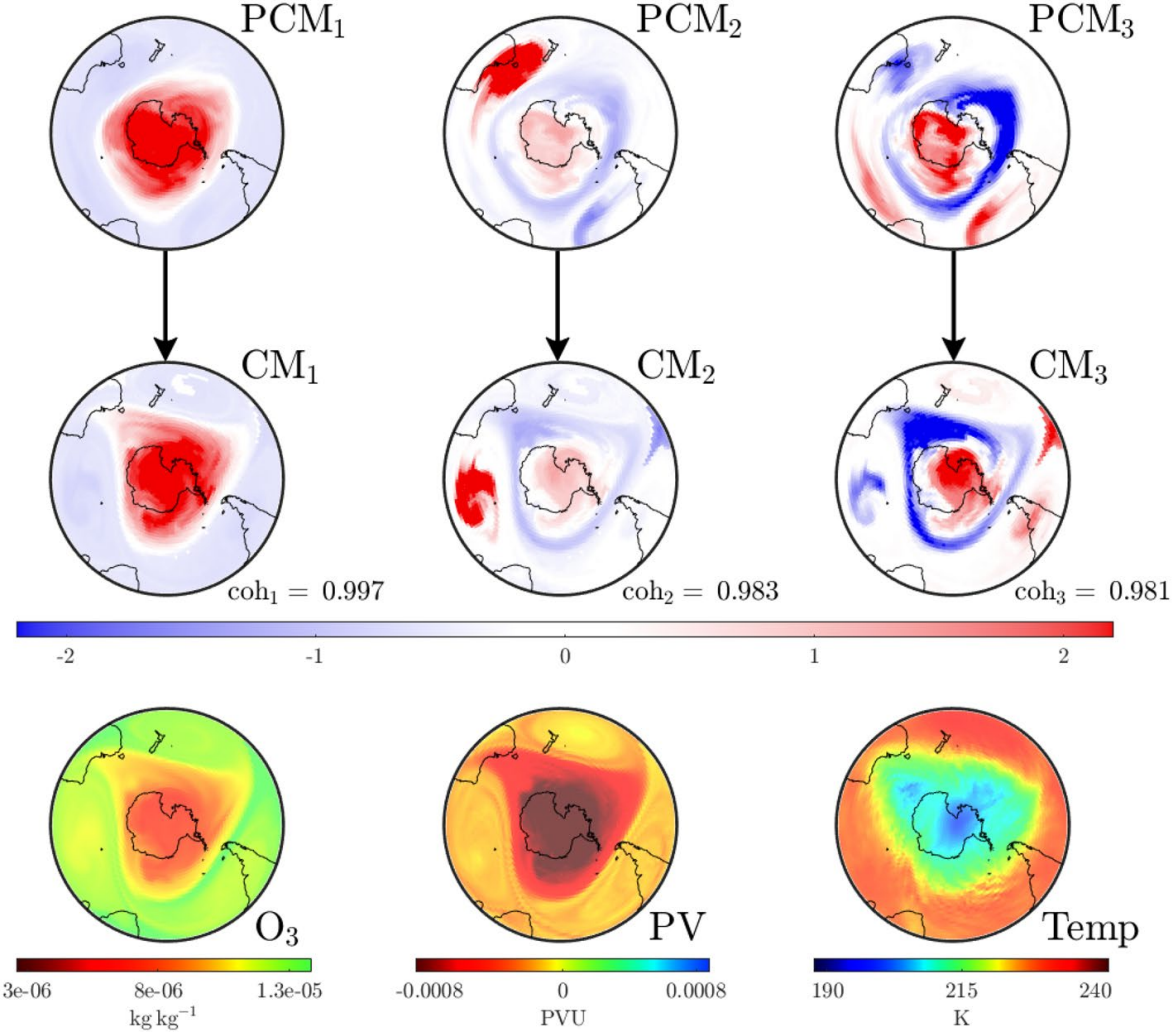
Plots on the $850\,\textrm{K}$ isentropic surface at UTC 0000 on August 17, 1999 for the area south of $20^{\circ }$ S. The bottom row shows data for atmospheric variables: ozone density ($\textrm{O}_3$, left), potential vorticity ($\textrm{PV}$, center) and temperature ($\textrm{Temp}$, right). The top and middle row show the leading three pre-convective modes (PCMs) and corresponding convective modes (CMs) along with their respective coherence levels, based purely on convective (wind) data for the preceding 2-week period. The interpretation is that the square of the PCM and CM fields represents how best to distribute the same total mass on the isentropic surface, such that when the corresponding air parcels on the PCM (on August 3) are pushed forward according to the wind velocities over a 2-week period, one gets approximately the CM field (on August 17). The mathematics of the “best matching” approach lead to PCM/CM modes, which are ordered according to their “coherence” ($\textrm{coh}$), indicating the goodness of matching. Observe how these purely convection (wind-data) based objects provide excellent predictors of the atmospheric variables: the SPV’s shape (red in the dominant mode, $\textrm{CM}_1$), the jet stream surrounding the SPV (blue triangular region in $\textrm{CM}_3$) and a high-pressure anti-cyclone in the Indian Ocean (red patch in $\textrm{CM}_2$).

In order to describe the results that follow, let us first expound the concepts of “convective modes” and “coherence” values. Figure 1 presents a snapshot of this information for our baseline case over the region south of $20^\circ$ S, on the $850\,\textrm{K}$ isentropic level in the atmosphere, at the time UTC 0000 on August 17, 1999. The lower row shows the ozone concentration (mass of ozone per kilogram of air), potential vorticity (potential vorticity units) and temperature (Kelvin) distributions at this instance in time. As described in “Supplementary Material” for this article, these variables are extracted from ECMWF ERA5 and NCEP CFSR/CFSv2 reanalysis products^14–16^. The middle row displays three convective modes ($\textrm{CM}$s) on August 17 of greatest coherence ($0< \textrm{coh} < 1$) with respect to wind data over the previous fortnight, obtained using methodology that will be described shortly. Notably, the only information used in this computation is the meridional (northerly) and zonal (easterly) component of wind on the $850\,\textrm{K}$ isentropic surface for the previous fortnight (beginning August 3) and the pressure distribution on that surface at the same time instances. The $\textrm{CM}$ with the highest coherence $\textrm{CM}_1$ is a remarkable predictor of the spatial variation of all three atmospheric variables pictured, notably the spatial shape of the SPV. The $\textrm{CM}$s with the next order coherence values also exhibit important spatial features, such as the jet region surrounding the SPV (blue in $\textrm{CM}_3$) and the anticyclone in the southern Indian Ocean (red in $\textrm{CM}_2$), traces of whose impacts are also visible in the other variables.

In addition to $\textrm{CM}$s, Fig. 1 displays pre-convective modes ($\textrm{PCM}$s) along the top row. These are paired with the $\textrm{CM}$s directly below. The intuition for these spatial structures is as follows. Suppose one is to decide how to distribute a given mass of air at both the initial time (August 3) and the final time (August 17, the date on which we desire information) on an isentropic surface, such that when the air mass at the initial time is pushed forward by the convective wind data, it most closely resembles the mass distribution that we have at the final time. While one might expect mass conservation to prevail on the surface, pushing air parcels forward based on the wind velocities usually results in some loss: velocity data is only available on a grid hence subgrid effects operate as numerical diffusion, filamented structures or large gradients are significantly impacted by diffusion, motion is only approximately confined to the isentropic surface, etc. Thus, there can only be an *approximate* matching between the pushed-forward initial air distribution and the total air mass distributed at the final time. The optimal pairings in this process are the pre-convective modes and convective modes (we cannot choose one of them independent of the other) each of which comes with a characteristic “coherence” value to quantify the quality of matching. Figure 1 illustrates three modes with the highest coherent values emerging from this process. The evidence is that “coherence with respect to the wind velocities” (in this case over the past fortnight) strongly impacts the variation of other atmospheric variables as well. Hence our usage of the word “convective”; our modes, based on wind velocities, describe how various quantities are convected.

Whilst $\textrm{PCM}$ and $\textrm{CM}$ fields are not standard distributions (observe from Fig. 1 that they have negative values), the *square* of their values at each location does correspond to a count of the *air parcels per unit area* locally on the isentropic surface. This is different from the *mass per unit area*. Mass is not distributed evenly on the isentropic surface and so pressure data—only required at the initial time—is used to perform an appropriate conversion via adiabatic gas theory. Regions of the $\textrm{PCM}$/$\textrm{CM}$ figures distinguished by darker colors signify a greater density of air parcels, their presence in these highly coherent modes indicates that *air masses in the darker regions are more robust to the wind velocities*. Hence one identifies coherent structures in these modes. Indeed, the dark reds in $\textrm{CM}_1$ of Fig. 1 get pushed to one another and identify the spatial location of the SPV at relevant times. Regions of dark blue are similarly highly robust. Regions whose color is close to white are transitional regions between the more robust structures. A detailed elucidation of the physical intuition, mathematics and algorithms that define these concepts is provided in the “Supplementary Material” document for this article.

The techniques we utilize build on well-established transfer operator methods^17–19^ that determine Lagrangian (going with the flow) coherent structures using only time-varying velocity data^20–22^. Whilst standard approaches seek partitions of space into distinct coherent structures^17–19^, our work tracks the spatial structure associated with the SPV whilst also identifying trends in the coherence levels of structures and signals that point to atypical events. This allows us to identify when certain structures are becoming less coherent. Moreover, we validate our findings throughout using independent observations of related variables; in this case, linking spatio-temporal convective effects with geophysical and chemical characteristics and their evolution.

The “Results and discussion” presented in the second section further formulates and applies the concept of convective modes and their coherence to the particular examination of *anomalous* occurrences (whereby the SPV exhibits unexpected or anachronistic behavior). Moreover, we show that the theory of convective modes and their coherence can be used to track the SPV, from its inception as a single vortical entity to the period of decay, which occurs as other structures acquire prominence throughout spring. The theory solidifies known connections between purely convective effects and the evolution of atmospheric variables. It does so by providing quantitative tools: numerical values of *convective modes* that allow for an understanding of their spatial distribution and temporal changes, and *coherence values* that indicate the likelihood of observing the spatial patterns of each convective mode. These tools enhance our understanding of the SPV’s coherence during its seasonal evolution and response to anomalous effects.

## Results and discussion

Our results are presented in several parts. In “Convective mode analysis across the years”, we provide a comparative analysis of the SPV across 4 years of interest (1999, 2002, 2019 and 2022). “1999: expected evolution” to “2022: accumulation of anomalistic occurrences” then address each year individually, placing greater emphasis on particular features and events relevant to a particular year.

### Convective mode analysis across the years

**Figure 2:**
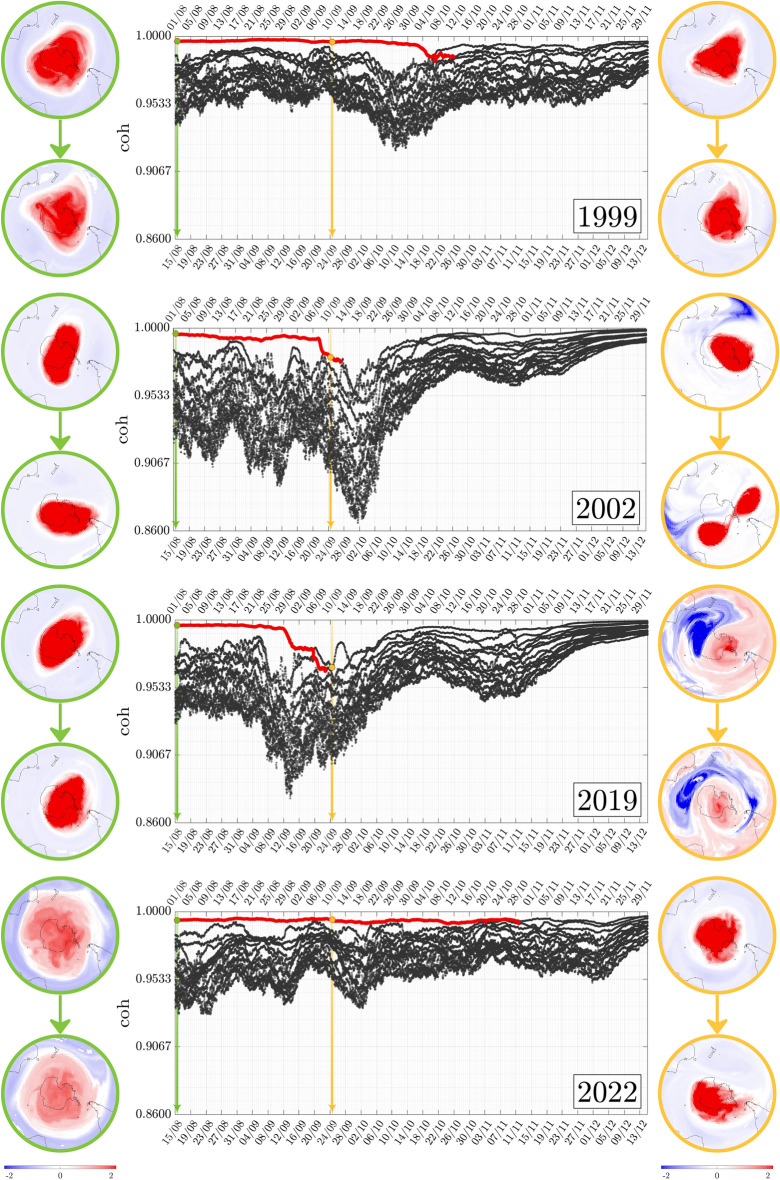
Composite figure of the evolution of the 14 highest coherence values across the four study years. The pre-convective and convective modes associated with two choices for each year (at times indicated by the green and gold lines and dots) are displayed on the left and right. Instances in which the SPV can be tracked as a single-vortex entity (the special convective mode) are indicated by coloring the coherence curves red. The SPV dissipates as a single entity at different times in the 4 years. Anomalous behavior is also observed in the early stratospheric warming years (2002 and 2019), with significant dipping in coherence values and atypical convective modes.

A broad overview of how convective modes and coherence values relate to the SPV across the four study years is presented as a composite figure in Fig. 2. All analyzed data sits on the $850\,\textrm{K}$ isentropic surface and the time-of-flow for the convection is uniformly set at 2 weeks.

Figure 2 consists of four horizontal panels, each of which represents 1 year of interest. The lower abscissa is the “current” date, while the upper abscissa is the “pre”-date, 2 weeks before the current date. The Convective Modes and their coherence are calculated with respect to wind velocities across the 2 week period, and the main plots indicate the temporal evolution of the fourteen highest coherence values. The evolution curves are shown in black, but specific parts of some curves are highlighted in red; the reason for this will be explained shortly. Convective modes associated with coherence values closest to 1 are the most prominent spatial structures anticipated from these wind velocities. In each plot, the Convective Mode with the highest coherence value and its accompanying pre-convective mode are shown for two instances in time: those associated with the green (August 15) and golden (September 24) vertical lines. The green and gold dots on the coherence value plots indicate *which* coherence value the chosen Convective Mode is associated with. For example, the two top left plots encircled in green are the Pre-Convective Mode (associated with August 1) and the Convective Mode it maps to (on August 15), which is expected to provide information on the atmospheric variables on August 15.

Many of the convective modes, pictured on the relevant dates (both at UTC 1200) for the 4 years, display a prominent SPV as a single-vortical entity. Both the pre-convective modes and convective modes for 1999, for example, indicate that the SPV maintains a single-vortex structure on these two dates. The green and gold dots are both on the most coherent, i.e., highest, curve. This emphasizes the fact that the SPV, as a single entity, is on these dates the most coherent structure as predicted from the wind data. We color parts of the coherence curves red if it corresponds to the SPV as a single-vortex entity in the $\textrm{PCM}$ figure, which we call the “tracked SPV” or “special convective mode” ($\textrm{SCM}$). In order to make this determination, we need to examine the $\textrm{PCM}$ plots, and track their changes with time. The methodology and algorithms underpinning this process are outlined in the “Supplementary Material” document that accompanies this article. Since the SPV ceases to exist as a coherent single vortical structure in the Austral spring, we expect the tracking to fail sometime around October. In 1999, our analysis from tracking the convective modes indicates that we fail to identify the SPV as a single structure around October 26; the coloring of the $\textrm{SCM}$ ceases on this date.

Indeed, the $\textrm{SCM}$ (red curve in Fig. 2) signals not only the past and current state of the SPV—it can also signal impending decay or geophysical anomalies. Instances in which there is a dominant coherence value that is well separated from the next highest ones indicate that the corresponding Convective Mode is significantly more dominant than other modes. For all years, early August is characterized by a leading coherence value (at around the 0.98 level) that is well separated from a jumble of other values near 0.95. This well-separated coherence value, identifiable as the SPV, fades in dominance as spring sets in. However, it does so in different fashions: in the “more standard” years 1999 and 2022, other coherence values gradually increase to close the gap, while in the sudden stratospheric warming (SSW) years 2002 and 2019, the dominant coherence value displays an untimely, dramatic drop. This occurs in late September in 2002 but somewhat earlier in 2019. Additionally, the coherence of other modes also drops substantially in comparison to 1999 and 2022. Hence, SSW events appear to be associated with there being less structures that exhibit spatial coherence in general. Moreover, the beginnings of a sharp drop in coherence of the Special Convective Mode is a potential precursor to the SPV dissipating.

The well-documented splitting of the SPV based on various atmospheric variables^23^ in 2002 is captured by the golden convective mode in Fig. 2. What occurs around the same time in 2019 is more nuanced, with the Special Convective Mode displaying *two* structures of *opposite* sign, indicating that there are two distinct co-evolving structures (in blue and red). Rather than splitting into two, the red SPV region is now influenced strongly by an adjacent sickle-shaped blue structure of similar size, which blocks the SPV from all directions except for the Atlantic. Both cases show a dramatic drop in the dominant coherence value in Fig. 2. Furthermore, the SPV is still identifiable as a single entity as the drop progresses. Indeed pictures of ozone, potential vorticity and temperature at such times do not necessarily indicate anything untoward. Remarkably, by the time a dramatic fall is complete, September 24 in each of these cases, the convective modes *do* show that the SPV no longer exists as a single entity; this is confirmed by pictures of the atmospheric variables in our subsequent analyses, “2002: significant splitting” and “2019: dramatic displacement”. Detailed animations of this event are also found in the Supplementary Material.

**Figure 3:**
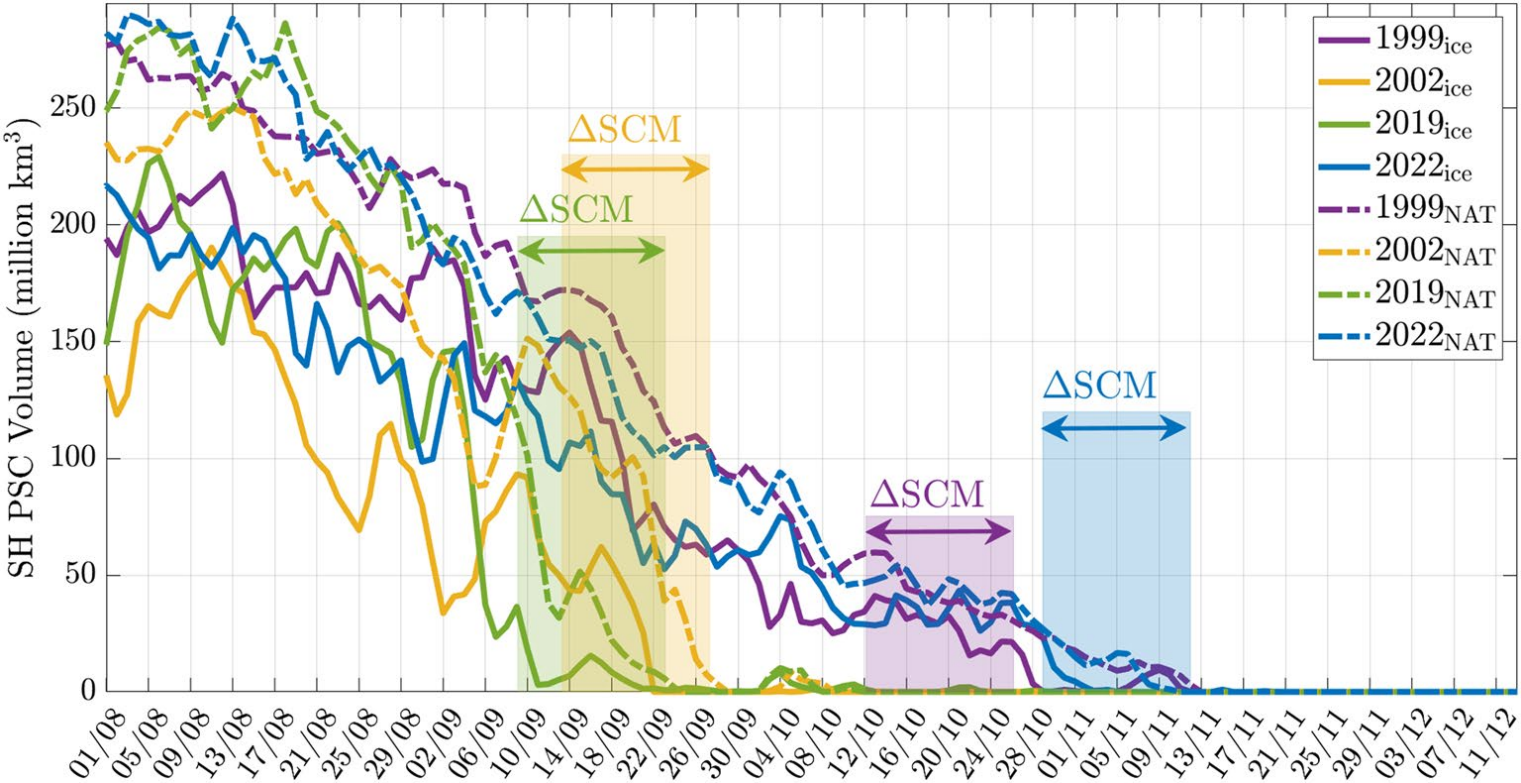
Polar stratospheric cloud (PSC) volume for the dates illustrated in Fig. 2. The time window $\Delta \textrm{SCM}$ is the period that immediately follows the final instance of the lifespan of the $\textrm{SCM}$ (red line in Fig. 2). The corresponding year is indicated in the plot legend (upper right corner). The subscript $\textrm{ice}$ indicates the volume of PSC formed by water vapor, whilst the subscript $\textrm{NAT}$ indicates PSCs formed by nitric acid trihydrate (NAT).

Evidence of a dissipation of the SPV, gleaned from the time evolution of the coherence values in Fig. 2, is consistent with an alternative measure: the volume of Polar Stratospheric Clouds (PSC) decaying towards zero. Figure 3 displays the decay of this quantity for each of the 4 years (from publicly available data^24^ obtained from the Modern-Era Retrospective analysis for Research and Applications, Version 2 (MERRA-2) assimilation). There are two measurements for PSC, one associated with the clouds formed by water vapor (indicated by the subscript $\textrm{ice}$) and one formed by nitric acid trihydrate ($\textrm{NAT}$). Overlaid on this plot are time windows from Fig. 2 associated with a breakdown in tracking that tells us the SPV is no longer identifiable. We call these intervals during which the Special Coherent Mode is no longer identifiable $\Delta \textrm{SCM}$. While these occur at different times each year, Fig. 3 demonstrates the remarkable consistency between our convective mode analysis and PSC data decaying to very small values, below around $20 \times 10^6 \, \textrm{km}^3$.

While Fig. 2 shows the evolution of coherence values for a multitude of $\textrm{CM}$s, it only pictures the dominant $\textrm{CM}$ at two instances in time. Obtaining additional insight requires knowledge of other $\textrm{CM}$s, in relation to their change in coherence with time. “1999: expected evolution” through “2022: accumulation of anomalistic occurrences” address the chosen years in further detail. In particular we demonstrate the structural changes experienced by the SPV as it dissipates and other $\textrm{CM}$s become more prominent.

### 1999: expected evolution

The time period from August through the start of December in 1999 is employed as a base case for comparing the expected annual behavior of the SPV. The establishment of a strong, coherent SPV by August was shown in Fig. 1. The SPV persists well into October 1999, as indicated in Fig. 2. This dominance is strengthened by the fact that its coherence value is well separated from lower values. We now analyze the eventual dissipation of the SPV using CMs.

**Figure 4:**
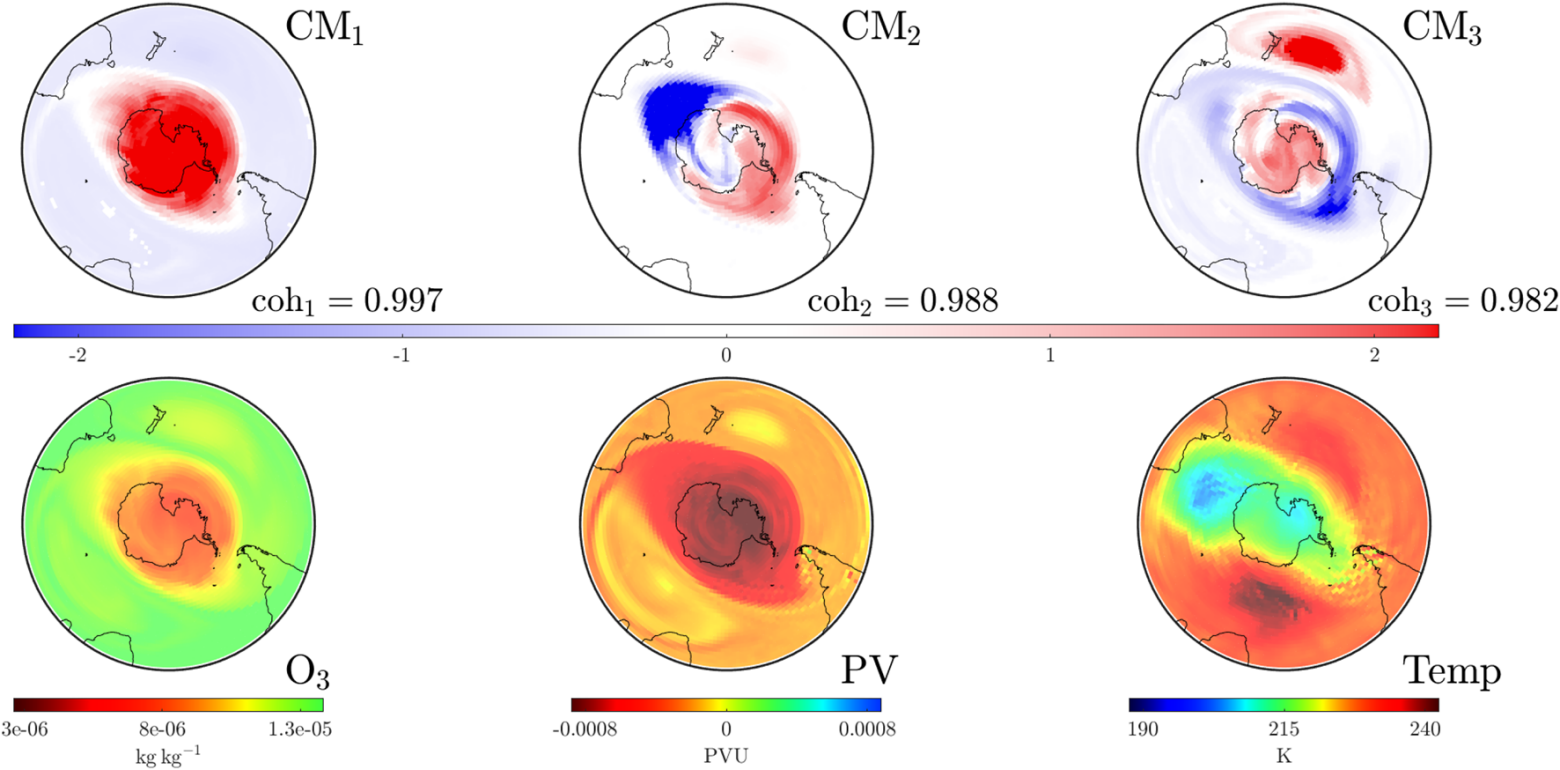
Convective modes (top row) and atmospheric variables (bottom row) at the $850\,\textrm{K}$ isentropic level on September 16, 1999 at UTC 0000. The dominant CM, $\textrm{CM}_1$, nicely captures the distribution of the atmospheric variables. $\textrm{CM}_3$ identifies a dynamically meaningful structure in the area near New Zealand associated with an unusually high number of blocking events this year.

By September 16 1999, it is clear from Fig. 4 that the dominant $\textrm{CM}$ still has the SPV structure with a well-separated coherence value. We note that $\textrm{CM}_3$ delineates the outer jet stream from the core it encases, but also detects a second structure east of New Zealand. Blocking in this region is characterized by a jet splitting. This coincides with observed blocking activity in the $120^\circ$ east–$150^\circ$ west meridian region during late winter^6^. We remark that Ozone depletion continued at an almost unprecedented rate over spring whilst summer also heralded a high number of blocking events in the $150^\circ$ east to $150^\circ$ west meridian region^25^. These observations agree with $\Delta \textrm{SCM}$ occurring well into October (Fig. 3) and our detection of various structures to the south of Australia and over New Zealand (Figs. 1, 4).

**Figure 5:**
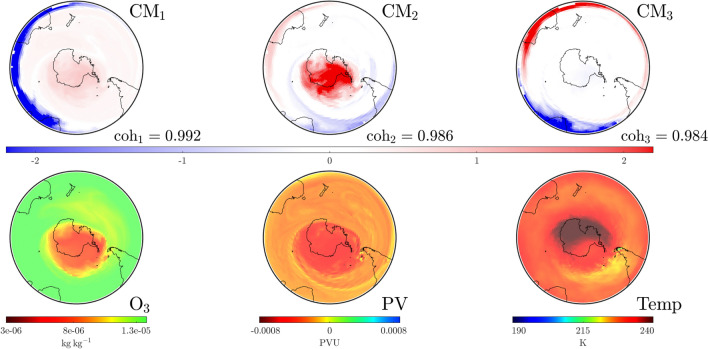
As in Fig. 4, but on October 25, 1999 at UTC 1200. The SPV is no longer the dominant CM, but has moved to $\textrm{CM}_2$. Warmer air in the stratosphere over Antarctica indicates that the SPV is dissipating, but lower levels of $\textrm{O}_3$ still persist.

Our analysis for October 25—near the end of the red tracked curve in Fig. 2 shows that the SPV has started to weaken and dissolve as detected (see CMs in Fig. 5). The SPV structure is now visible only in $\textrm{CM}_2$ with $\textrm{CM}_1$ indicating that influential structures are moving in from outside the domain of interest, i.e., from the subtropical regions. The temperature above Antarctica, close to the center of the structure above the pole, is now warmer than at higher latitudes. This signals that, by the temperature-based characterization, the SPV is indeed dissipating. This agrees with our CM analysis that detects not only the dissipation of the SPV—it also provides insight into how dissipation occurred via the increasing dominance of other modes. In the Supplementary Material we provide a video of the temporal evolution of these $\textrm{CM}$s and atmospheric variables for 1999. Videos are also provided for the other years we study: 2002, 2019 and 2022.

### 2002: significant splitting

**Figure 6:**
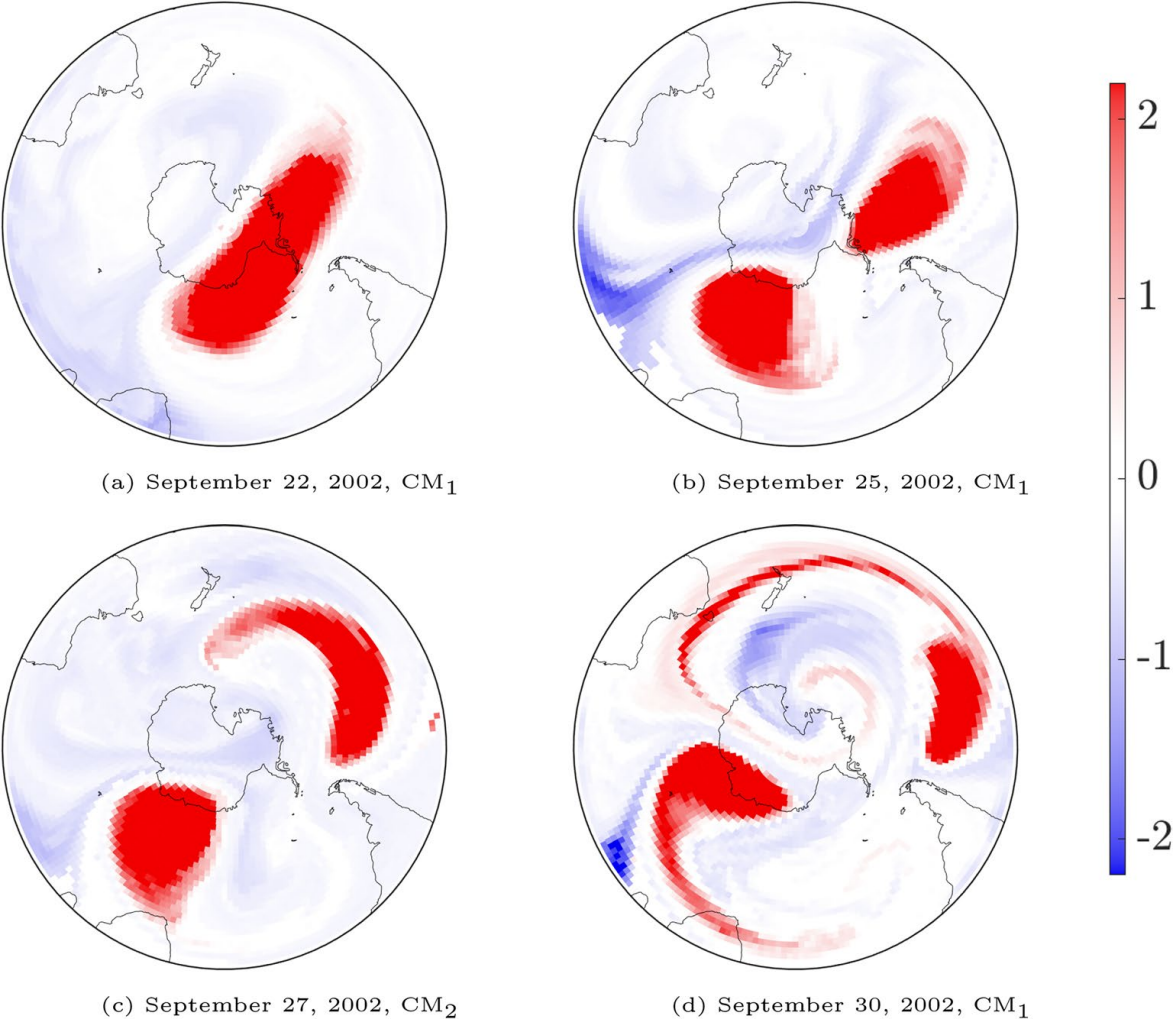
Important convective modes on the $850\,\textrm{K}$ level at UTC 1200 at several dates associated with the 2002 splitting event. A direct comparison with Fig. 7 of Charlton et al.^1^ shows how the theory of $\textrm{CM}$s, computed from wind velocities over the past 2 weeks, gives an excellent predictor of the spatial distribution of Ertel’s PV on the relevant dates.

The fall in coherence of the 2002 tracked $\textrm{PCM}$ in Fig. 2 corresponds to the initiation of an SSW event that culminated in a dramatic separation of the SPV^23^. By September 22 the SPV had become elongated and somewhat primed for splitting^1^. Figure 6a hints at an anticyclone, characterized by low $\textrm{PV}$, sitting south of Australia, enhancing the mixing of filaments from the outer SPV with air from the mid-latitudes. This, in combination with a weaker anticyclone near the tip of South America, encouraged the elongation and subsequent separation of the SPV into two cyclonic vortices by September 25^1^, as shown in Fig. 6b. The efficacy of our $\textrm{CM}$ analysis presented in Fig. 6, is further proven via direct comparison to results presented in Figure 7 of Charlton et al.^1^, which plots Ertel’s $\textrm{PV}$ at the $850\,\textrm{K}$ level over an 8 day period.

**Figure 7:**
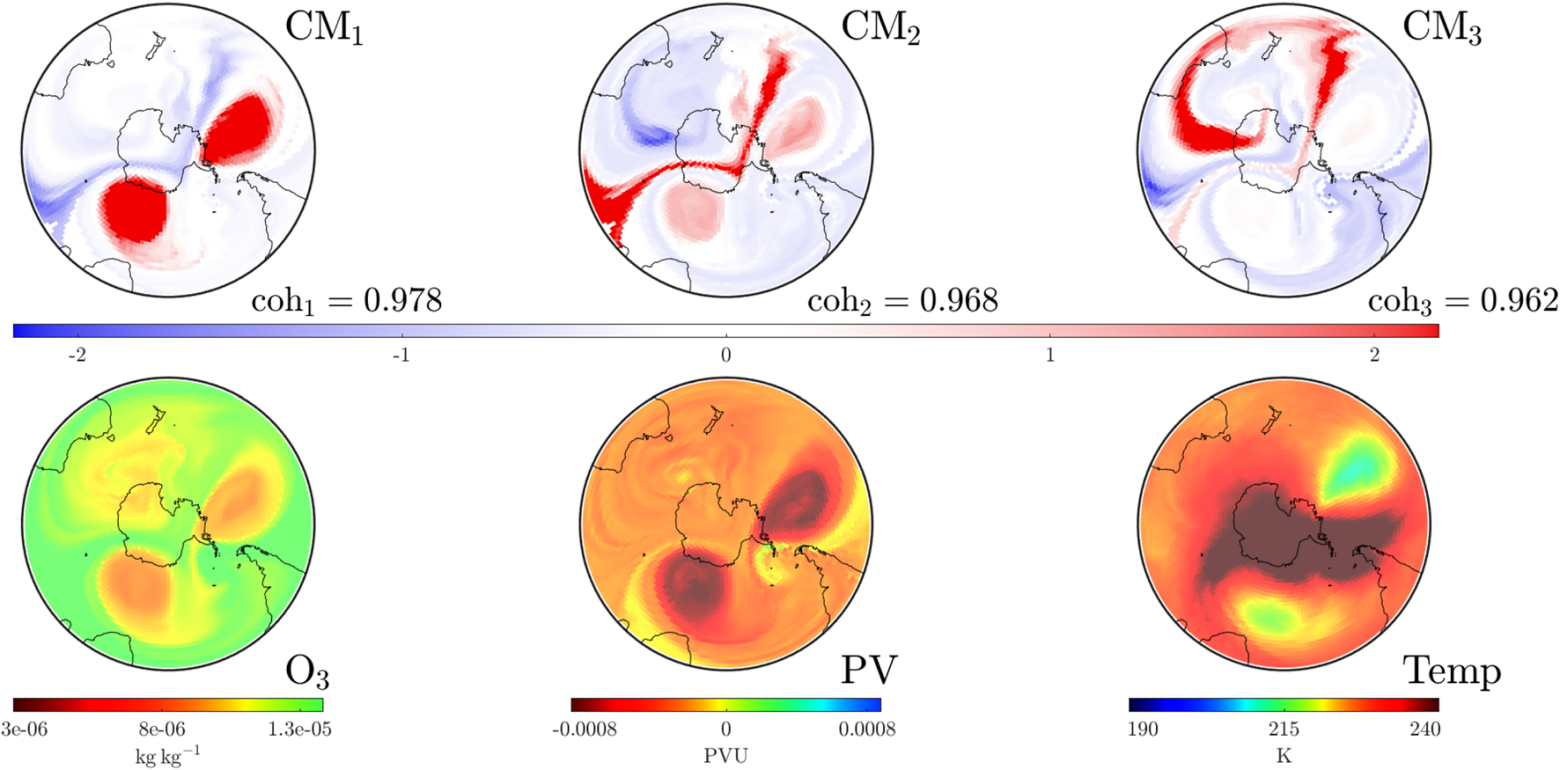
As in Fig. 4, but on September 26, 2002 at UTC 0000. $\textrm{CM}_1$ shows that the two structures the SPV has split into, while the “separating” red filament in $\textrm{CM}_2$ hints at a structure which contributed to this split, and may be associated with an incoming temperature front from the subtropics. The additional ozone depleted anticyclone south of Australia^1^ is captured in $\textrm{CM}_2$, while the “separating” red filament in $\textrm{CM}_3$ indicates its strong separation from the subtropical stratosphere while being less separated from the lower $\textrm{O}_3$ polar region.

To investigate the role of other $\textrm{CM}$s in this break-up, we examine the top three $\textrm{CM}$s on September 26, shown in Fig. 7. The dominant $\textrm{CM}_1$ displays the split SPV, at a coherence level of 0.978. $\textrm{CM}_2$ of coherency $\textrm{coh}_2= 0.968$ shows a “tongue” of striking stratospheric air drawn pole-ward from the mid-latitudes. This corresponds to a more dramatic example of earlier findings that highlight the role of air from the subtropics in structural disturbances^26,27^. $\textrm{CM}_3$ highlights a (red) shield from the subtropics eschewing the third patch of lower $\textrm{O}_3$, once connected to the polar ozone hole.

As shown in Figs. 2 and 3, the splitting of the SPV is associated with the downfall of the tracked $\textrm{PCM}$ (as a single vortex entity). The center plot for the year 2002 of Fig. 2 shows a rapid decrease in the associated characteristic coherence values from $\textrm{coh}_1=0.992$ on September 20 to $\textrm{coh}_1=0.978$ on September 26. The tracking (in red) of the $\textrm{PCM}$ as a single vortex comes to an end shortly after.

### 2019: dramatic displacement

**Figure 8:**
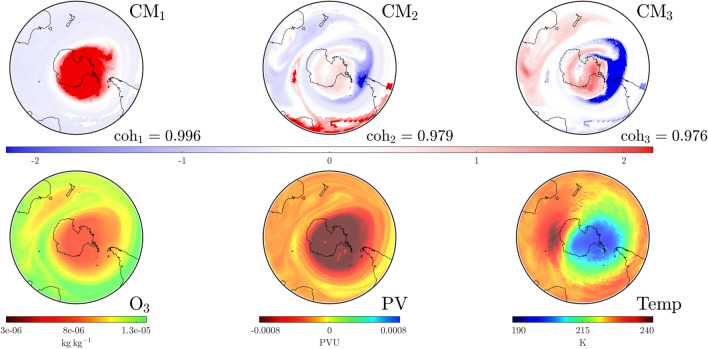
As in Fig. 4, but on August 20, 2019 at UTC 1200. At this time, the dominant CM, $\textrm{CM}_1$, has high coherence and shows a well-established SPV. Signatures of the anticyclonic region south of Australia are emerging in the subdominant CMs, and visible in the $\textrm{O}_3$ distribution.

**Figure 9:**
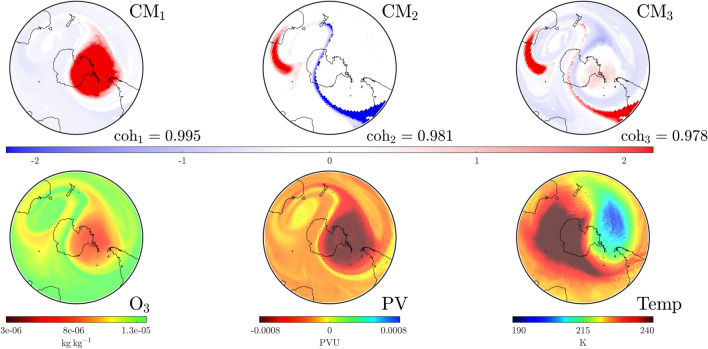
As in Fig. 4, but on September 7, 2019 at UTC 0000. The subdominant CMs display filaments indicating how subtropical air is pulled across Antarctica, with the filaments (lying along boundaries of the SPV) pushing the SPV towards South America.

**Figure 10:**
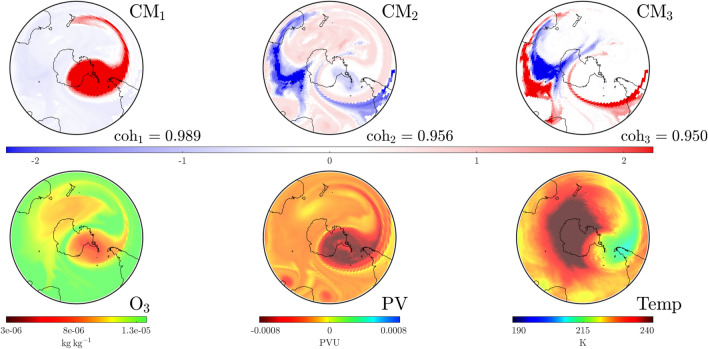
As in Fig. 4, but on September 13, 2019 at UTC 0000. The SPV exists as a “comma-like” structure in $\textrm{CM}_1$, but the more complex subdominant $\textrm{CM}$s illustrate structures which are leading towards the SPV’s movement and dissipation.

**Figure 11:**
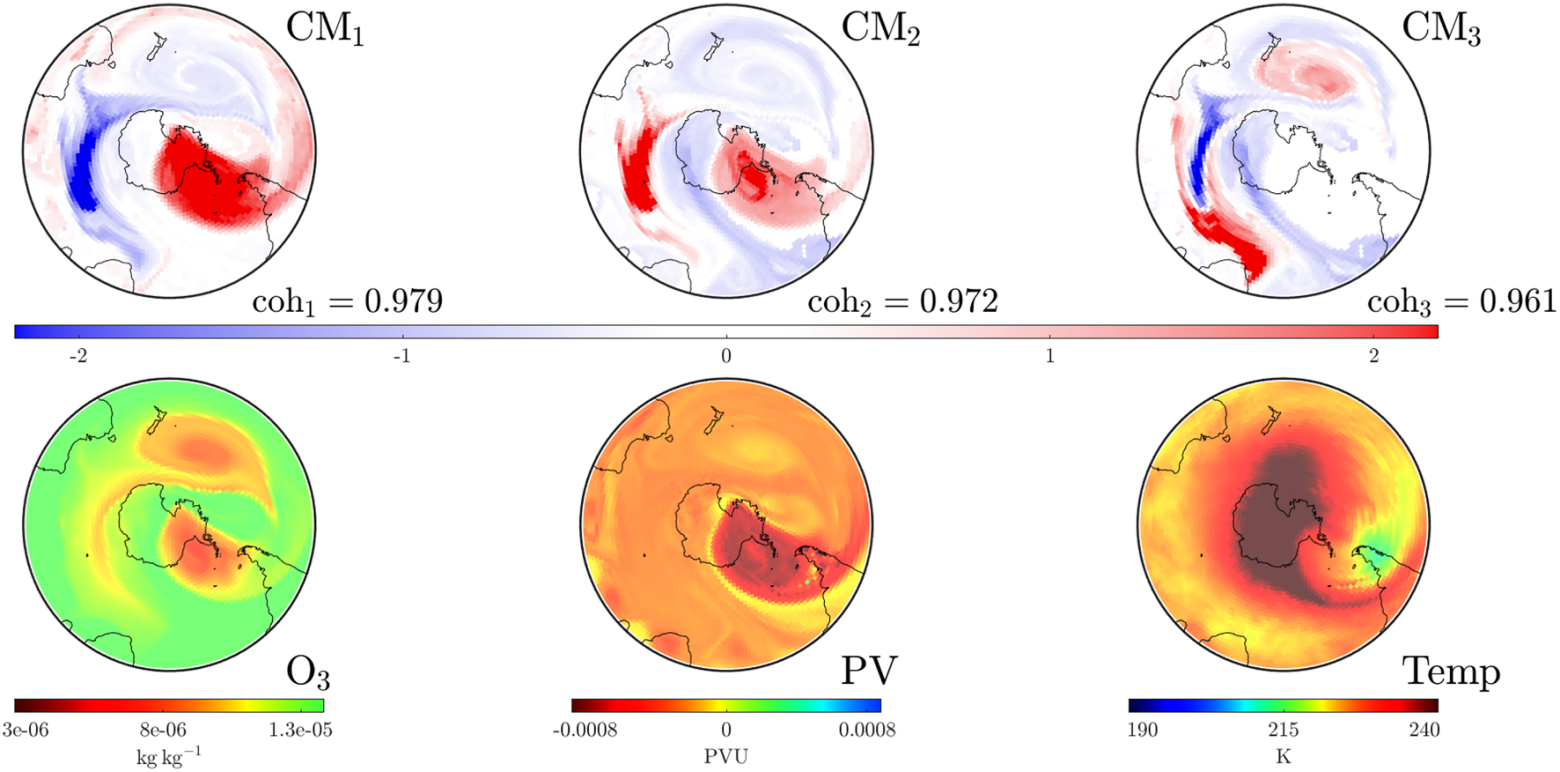
As in Fig. 4, but on September 19, 2019 at UTC 1200. A single-vortex SPV is less apparent, coherence values indicate that $\textrm{CM}_1$ is less dominant, and the $\textrm{CM}$s display a variety of complicated features which are present in the atmospheric variables.

The year 2019 saw another SSW event. Detailed meteorological and geophysical assessments of this event are available^2,9–13^, but a full understanding of the events is still developing^11^. By September, the ozone hole was noticeably smaller in comparison to recent records^28–30^. The warming culminated with a weakening of the polar vortex at 10hPa on September 11^31^ and whilst this SSW did not result in a splitting, evident changes in the behavior of the SPV are explored in detail using $\textrm{CM}$s in Figs. 8, 9, 10 and 11.

On August 20 (Fig. 8), the dominant $\textrm{CM}$ is the SPV. $\textrm{CM}_2$ and $\textrm{CM}_3$ show stratospheric developments over southern Australia and New Zealand; this comprises an elongated anticyclonic structure that matches the upper ozone patch in the lower panel. Moreover, $\textrm{CM}_2$ highlights a train of activity from South America to the tip of Africa. One particular filament from this region is pulled around Antarctica, feeding in between the cyclonic SPV and the anticyclonic region south of Australia. This is not unlike the dynamics reported to have occurred during 2017^32^, in which case unusual activity in mid to late August formed stratospheric anticyclones capable of drawing in filaments from the tropics along the outer border of the SPV. By September 7 (Fig. 9), this filament is much more dramatic. Shown in blue in $\textrm{CM}_2$, it extends from the subtropical Americas across Antarctica to New Zealand. The filament borders the SPV, which is consequently pushed towards South America ($\textrm{CM}_1$). These findings are supported by previous research observing the easterly translation taken by a filament or “tongue” of tropical stratospheric air^26,27^.

Coherence values of the subdominant $\textrm{CM}$s are increased from their values on August 20. Furthermore, $\textrm{CM}_3$ indicates the establishment of complex behavior across the Southern Hemisphere. The importance of other features from the $\textrm{CM}$s—such as the red “tongue” of $\textrm{CM}_2$ and $\textrm{CM}_3$ emanating from Australia—also predict spatial structures in the atmospheric variables. The evolution of temperature and geopotential height at 10hPa has been studied previously^31^, finding that high temperatures stretched toward the South Pole between September 6 and 8 without quite reaching it. Our analysis for September 13 (Fig. 10) shows that the dominant $\textrm{CM}$ acquires a “comma-like” structure that is pushed towards South America, consistent with the structures visible in the $\textrm{O}_3$, $\textrm{PV}$ and $\textrm{Temp}$ observations. This shape is characteristic of displacement events that occur due to the shearing of vortices from the SPV^23^. By September 19 it is increasingly difficult to identify the SPV (Fig. 11). Indeed, the SPV appears to be shifting to $\textrm{CM}_2$, and the coherence values for $\textrm{CM}_1$ and $\textrm{CM}_2$ are now significantly closer. One recalls the dramatic fall of the largest coherent value (Fig. 2) and the fact that multiple coherence values (corresponding to different $\textrm{CM}$s) are close at this time. Hence the SPV as a single vortex has dissipated but the $\textrm{CM}$ plots and their coherence allow us to analyze the temporal evolution of spatial structures that lead to this dissipation.

### 2022: accumulation of anomalistic occurrences

The year 2022 was characterized by the prolonged impact of the 2019 Australian wildfires and the Hunga Tonga-Hunga Ha’apai eruptions. The wildfires of 2019/2020 were correlated with noticeable changes throughout the stratosphere in both 2020 and 2021, during which uncharacteristically large and persistent ozone holes were evident^33,34^. Nevertheless, our understanding of the ability of aged smoke particles to influence Polar Stratospheric Cloud formation remains incomplete^35–37^. Conversely, the eruption of shallow water volcanoes Hunga Tonga-Hunga Ha’apai sent large amounts of aerosols well into the upper stratosphere whilst also increasing the presence of water vapor and sulfates. Studies suggest this vapor will inhabit regions near the SPV through to at least October 2022^38,39^, alter surface temperatures and lead to long term implications for stratospheric ozone. Further monitoring and assessment is required to better quantify the implications of this eruption^40–42^.

**Figure 12:**
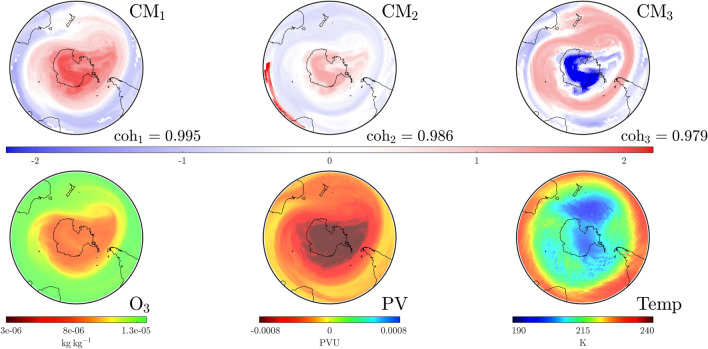
As in Fig. 4, but on August 20, 2022 at UTC 1200. The SPV, as identified via $\textrm{CM}_1$ and the temperature, is substantially more diffuse and extended than it was on the same day 3 years previously (see Fig. 8).

Whilst understandings of the impact of these events gradually increases, we are in a position to analyze recent data using our $\textrm{CM}$ techniques. A direct comparison of $\textrm{CM}_1$ from Figs. 8, 12 shows that in 2022 the SPV dominated a larger region on August 20 than it did on the same day in 2019 (prior to the SSW event). It is also clear from Fig. 12 that the temperature of the stratosphere on the $850\,\textrm{K}$ surface was less zonally concentrated than in 2019. As in Fig. 8, $\textrm{CM}_2$ of Fig. 12 shows filaments from the sub-tropical regions attempting to penetrate the higher latitudes of the $850\,\textrm{K}$ surface. However, in contrast to the 2019 case, the lack of strong anticyclonic activity south of Australia means these filaments are not pulled across the Antarctic region at this time.

**Figure 13:**
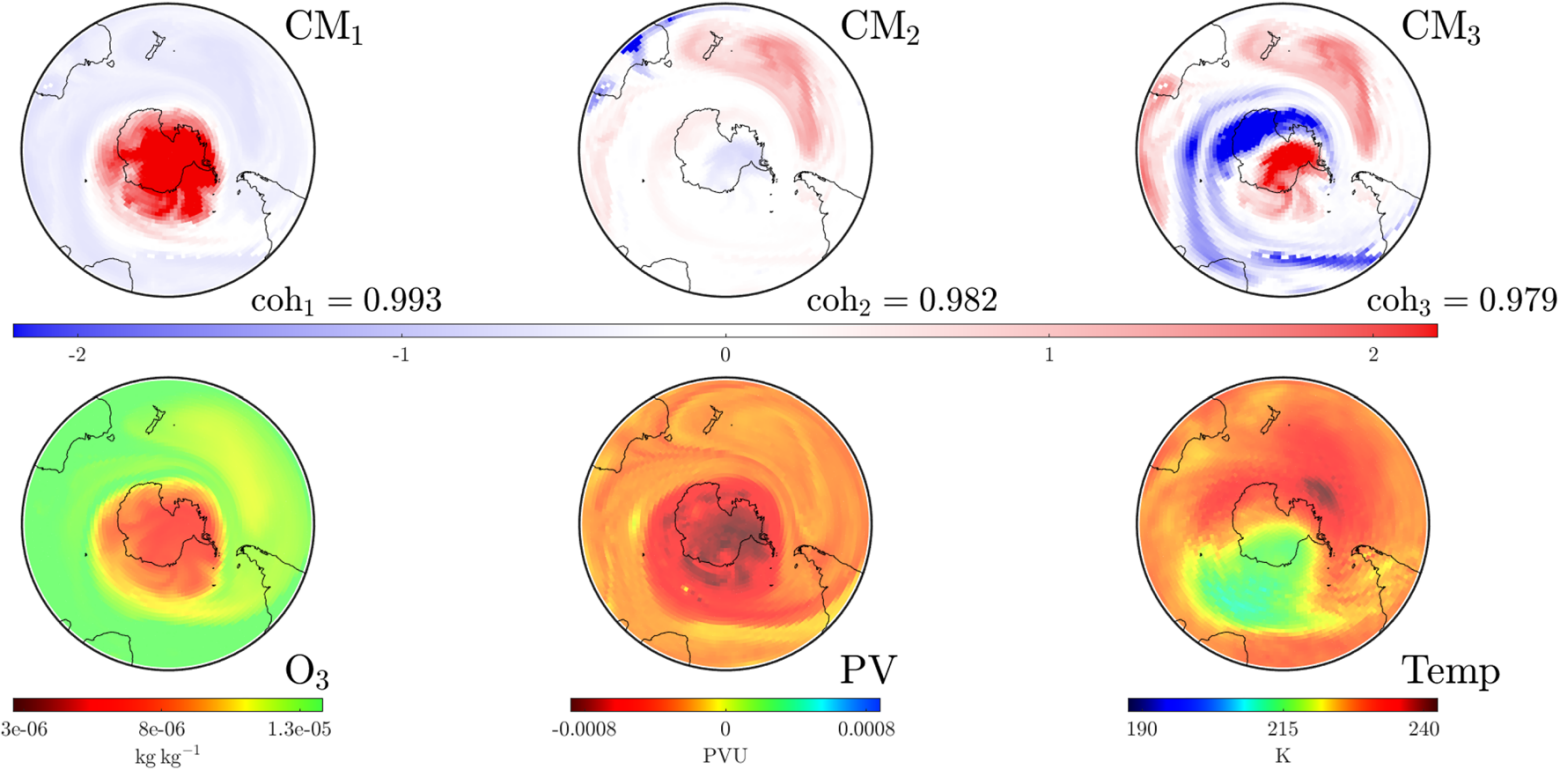
As in Fig. 4, but on September 26, 2022 at UTC 0000. The dominant $\textrm{CM}$ has become more compact that on August 20 (Fig. 12) due to shearing effects from lower latitudes, as visible in $\textrm{CM}_3$. Activity in the southern Pacific visible in $\textrm{CM}_3$ is bringing in warmer air, pushing the lower temperature air mass towards southern Africa.

**Figure 14:**
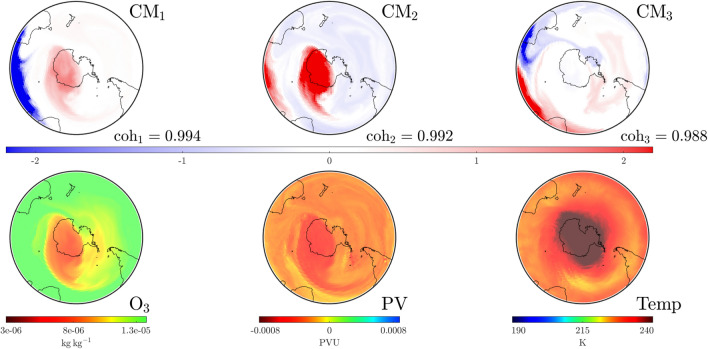
As in Fig. 4, but on November 13, 2022 at UTC 0000. The dominant $\textrm{CM}$, $\textrm{CM}_1$ now resembles a single-vortex SPV less than the subdominant $\textrm{CM}_2$. Thus, the SPV, as identified via the $\textrm{CM}$s and as consistent with the $\textrm{O}_3$ data, persists for longer in 2022 than in the other study years, but in a less coherent state.

Shearing effects from the lower latitudes lead to a more compact $\textrm{CM}_1$ by September 26, as shown in Fig. 13. (On this same day in 2002, $\textrm{CM}_1$ experienced splitting behavior.) In 2022, the SPV remained a coherently evolving structure characterized by a region of air that remained cooler than the external environs. This persistence is reinforced by the (red) SPV tracking results in Fig. 2; the 2002 tracking ceased near this day, while that for 2022 continues for a considerable amount of time. However, one can see from $\textrm{CM}_3$ in Fig. 13 that “tongues” from the subtropical Atlantic and Indian regions are swirling around the SPV (blue), with a strong anticyclonic region (red) pulling in warmer air from the southern Pacific, pushing the cooler-air region towards Africa. By November 13, the temperature in Fig. 14 demonstrates that warmer air has moved in and the polar region is now comparatively hotter than surrounding regions. The dominant $\textrm{CM}$ is beginning to look less like the SPV, while $\textrm{CM}_2$ is acquiring its spatial characteristics, albeit at a weakened level. The tracking of the SPV in the comparative Fig. 2 shows that the red curve is now associated with the *second* rather than the first coherent value. Thus, there is now a spatial structure, $\textrm{CM}_1$ that is more dominant than the SPV (Fig. 14) but the SPV persists for much longer than in other years (see Fig. 2). This is consistent with an uncharacteristically persistent ozone hole for the same year.

## Conclusions

Throughout this article, we developed the method of convective modes—distinct spatial structures that can be intuited as distributions of air parcels being pushed by wind velocities over a certain time period. We chose a time period of 2 weeks, and limited our analysis to the $850\,\textrm{K}$ isentropic surface above the Antarctic region to assess the stratospheric Southern Polar Vortex. Each Convective Mode—i.e., each spatial distribution—is associated with a coherence value, indicating how likely that spatial distribution is to be observed. Convective modes are derived from wind velocity data *only*, except for an area-to-mass conversion associated with pressure data at the initial time. Their computation does not use data such as temperature, potential vorticity, ozone, or polar stratospheric cloud information. Nonetheless, the convective modes were shown to be excellent predictors of these atmospheric variables, indicating how dominant convection is as a mechanism for their distribution.

Instances in which the convective modes do not mirror the spatial structures of some variables can occur if the initial distribution of the variable is quite different from what is expected from winds alone, or if non wind-related physical effects are particularly strong over the time period. In either case, while winds do attempt to mould the variable’s spatial distribution to preferred states, i.e., the convective modes, other conditions/effects may be battling against it. Many of the temperature distributions we have shown, for example, are not exactly shaped by the convective modes, reflecting the fact that other effects that are not completely linked to the winds (equatorial warming, say) are in operation. However, convective modes do effectively predict the spatial distributions of the atmospheric variables considered.

The convective modes we define here are based on the transfer operator method, which has been successfully developed in the assessment of Lagrangian coherence in assorted geophysical flows^17–19^. In contrast with many of those studies, our goal has not been to separate flows into coherent regions, but rather to obtain (potentially complicated) spatial structures and to study their persistence, as measured by how the coherence values we ascribe to them change with time. For example, we can track the persistence of the SPV over time, and study how influential structures, associated with other phenomena, eventually break it apart. In anomalous situations, such as when there is sudden stratospheric warming, we see that convective modes bearing different spatial structures than our usual expectation acquire prominence. Some examples of these structures are those associated with anticyclones in the southern Pacific, “tongues” emanating from subtropical regions, and distortions of the jet stream surrounding the vortex. As the coherence values of each spatial structure vary with time, one can easily characterize which structures are weakening and which are strengthening.

Recognizing sharp drops in the coherence value of the special convective mode (as in 2002 and 2019 in Fig. 2) allows one to identify potential precursors to the SPV dissipating in unexpected ways. By tracking other convective modes, whose coherence values are concurrently increasing relative to that of the SPV, we can moreover predict the spatial ways in which the SPV is poised to break up.

The convective modes technology allows one to thoroughly examine how spatial structures evolve in atmospheric flows via convection. To combat the need for specialized mathematical knowledge, often associated with transfer operator techniques, we have provided a step-by-step description of both the process and physical justification for why this method works (see “Supplementary Material”). These techniques are easily adapted to more general analyses and we have aimed our research at enabling practitioners to implement this methodology, and its extensions, for the analysis of evolving coherent structures in (bio)geophysical flows.

### Supplementary Information

Supplementary Information.

Supplementary Video 1.

Supplementary Video 2.

Supplementary Video 3.

Supplementary Video 4.

Supplementary Legends.

## Data Availability

The data used in this study is publicly available from the European Centre for Medium-Range Weather Forecasts (ECMWF), National Centers for Environmental Prediction (NCEP) and National Aeronautics and Space Administration (NASA) Goddard Space Flight Center Ozone Watch.

## Code availability

Computations and illustrations were done using MATLAB with assistance from the GAIO software package^43^. All code and constructed data sets are available from the corresponding author on reasonable request.
