# Supplementary material for: Convective modes reveal the incoherence of the Southern Polar Vortex
Source: Sci Rep. 2024 Jan 10;14:966. doi: 10.1038/s41598-023-50411-x (PMC10782018; doi:10.1038/s41598-023-50411-x)
Supplement: Supplementary file 1 — Supplementary Information. [file 41598_2023_50411_MOESM1_ESM.pdf]

Supplementary Material for "Convective modes reveal the incoherence of the Southern Polar Vortex"

Chantelle Blachut[1*] and Sanjeeva Balasuriya[1]

[1*]School of Computer and Mathematical Sciences, University of Adelaide, Adelaide, SA 5005, Australia.

*Corresponding author(s). E-mail(s): chantelle.blachut@adelaide.edu.au;
Contributing authors: sanjeevabalasuriya@yahoo.com;

1 Methods

1.1 Data

All cases studied throughout this paper are analysed on an 850 K isentropic surface. The corresponding data was obtained from three main sources. Our predominant source was the ERA5 atmospheric reanalysis data produced by the Copernicus Climate Change Service at the European Centre for Medium-Range Weather Forecasts (ECMWF) [1]. ERA5 data is available at a resolution of 0.25° in both the longitudinal and latitudinal directions and hourly in the temporal direction. This gives $1440 \times 361 \times 3265$ data points in the respective dimensions for the time period UTC 0000 August 1 to UTC 0000 December 15 for each of the years 1999, 2002, 2019 and 2022. In particular we utilise the Eastward and Northward components of wind (ms^{-1}) as well as the pressure (variable Pres with units Pa), mass of ozone per kilogram of air (variable O_3 with units $\mathrm{kg\,kg}^{-1}$) and potential vorticity (variable PV with units PVU where $1\,\mathrm{PVU} = 10^{-6}\mathrm{Km}^2\mathrm{kg}^{-1}\mathrm{s}^{-1}$).

Temperature (variable Temp with units K) on the 850 K isentropic surface was obtained from the National Centers for Environmental Prediction (NCEP) Climate Forecast System Reanalysis (CFSR) [2] for the years 1999 and 2002 and from NCEP Climate Forecast System Version 2 (CFSv2) [3] for the years

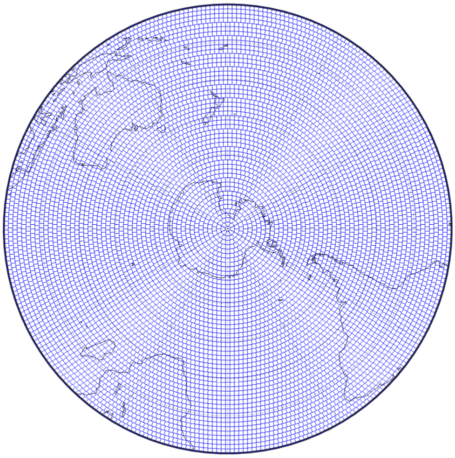

Supplementary figure S1: Graphical representation of partition of the full southern hemisphere into $d = 8073$ cells.

2019 and 2022. CFSR products are available at 0.3° horizontal resolutions and CFSv2 are available at 0.2°. The 6-hourly products are initialized daily at UTC 0000, 0600, 1200, and 1800.

The third component of data employed in this paper is Polar Stratospheric Cloud (PSC) volume formed from either nitric acid trihydrate (NAT) or water vapour (ice) constituents. Daily volume for the years 1999, 2002, 2019 and 2022 was generated using the Modern-Era Retrospective analysis for Research and Applications, Version 2 (MERRA-2) assimilation, produced by the Goddard Earth Observing System Data Assimilation System (GEOS DAS). This data is publicly available from National Aeronautics and Space Administration (NASA) Goddard Space Flight Center Ozone Watch [4].

1.2 Identifying Convective Modes and their coherence

Here, we describe the numerical procedure that we follow in order to compute the Convective Modes and their coherence. This is associated with a broader class of methods ("Lagrangian coherent structures") which seek coherence of fluid parcels following the Lagrangian flow [5–7]. Our work builds on that of many authors who have used the transfer operator approach which provides insight into coherence [8–13]; however, there are some differences. Rather than simply listing a numerical procedure, we provide a careful step-by-step description of both the process and the rationale behind it. To achieve our goal of having an intuitive description that is accessible to practitioners, we have consciously sidestepped deeper mathematical constructs – related for example to ergodic theory, infinite-dimensional operators, or function spaces with weighted inner products – which many related articles use. Occasional commentary is provided for readers who may be interested in these concepts.

Consider the problem of determining convective modes associated with motion on an isentropic surface in the atmosphere from an initial time t to a final time which is a time τ after the initial time. We will describe our approach in point-form, providing intuition for each step of the process. We refer readers interested only in the summary of this process to Algorithm 1.

1. We first partition the 850 K isentropic surface above the Southern Hemisphere into roughly equally sized cells as shown in Figure S1. We have $d = 8073$ cells in this partition, which is based on a method suggested by [14] that uses approximately equal lengths in the meridional direction regardless of latitude.
2. As our initial time cells, we consider the part of the Southern Hemisphere south of approximately $-20°$ degrees which comprises $m = 4949$ cells. We label these cells as $\{B_i\}_{i=1}^{m}$.
3. We want to determine the *transfer operator* [11, 15–23] which pushes forward a distribution of air at the initial time t to the final time $t + \tau$. To do so, we think of the transfer (Perron-Frobenius) operator as an *Ulam matrix* [11, 15, 23] using the following standard approach. (The transfer operator is usually defined over continuous space – rather than over cells – and operates on continuously-defined distributions. The Ulam matrix, being a transfer from m to n cells, is a *finite-dimensional* matrix approximation to the (theoretical) infinite-dimensional transfer (Perron-Frobenius) operator. Since our data is inevitably on a grid, we simply express everything in the finite-dimensional setting to avoid unnecessary mathematical complications.) In each cell, we seed $Q = 100$ particles uniformly, and then follow the flow for a time τ. In other words, for each such particle $\boldsymbol{x}_0$ we numerically solve
$$\frac{\mathrm{d}\boldsymbol{x}}{\mathrm{d}s} = \mathbf{vel}(\boldsymbol{x}, s) \quad ; \quad \boldsymbol{x}(t) = \boldsymbol{x}_0 \quad ; \quad s \in [t, t+\tau], \tag{1}$$
where **vel** is the time-varying two-dimensional Eulerian velocity data on the isentropic surface, and determine the final location $\boldsymbol{x}(t+\tau)$. We solve this using a second-order Runge-Kutta algorithm with timestep $h = 1$ hour consistent with the data, and need to interpolate the velocity data during this process, since it is only available on a grid. Let $\{C_j\}_{j=1}^{n}$ be the collection of cells from the original d cells on which particles eventually land. It is possible that some particles will venture north of the $-20°$ parallel, and also that not every one of the $\{B_i\}$ cells will be landed back on. So in general, $n \neq m$, and is determined by the data. While there will be many cells which are in both sets $\{B_i\}_{i=1}^{m}$ and $\{C_j\}_{j=1}^{n}$, they need not have identical labelling, that is, B_2 may refer to the same cell at C_4, for example. By counting the proportion of particles that were seeded in cell B_i and which arrive in a cell C_j, we form the probability $P_{i,j}$ of transition from B_i to C_j, i.e.,
$$P_{i,j} = \frac{\#\text{ times that } \boldsymbol{x}(t+\tau) \in C_j \text{ given that } \boldsymbol{x}(t) \in B_i}{Q}. \tag{2}$$

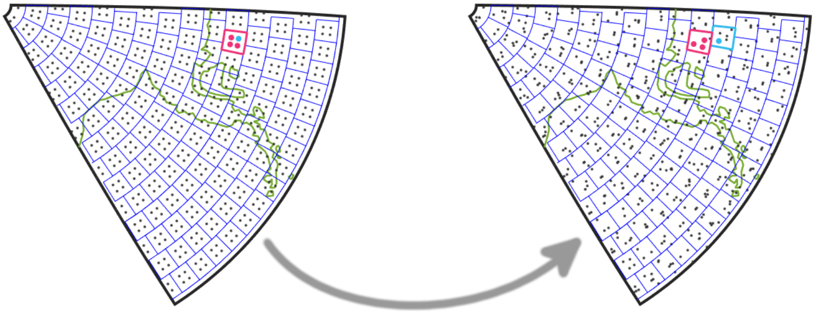

Supplementary figure S2: An illustration of how the transfer operator $\boldsymbol{P}$ is computed. A uniform collection of of $Q = 100$ initial conditions are seeded per cell, but $Q = 4$ is pictured here for clarity. The cell in which each seed lands at time $t + \tau$ is noted and the conditional probability of transitioning between cells is calculated. In this illustrative example, suppose the cell in which the four colored (comparatively thicker) initial conditions are placed is B_{57} (magenta cell), and the final cells to which these go are C_{23} (the same cell as B_{57}, but the labelling will in general be different for the final cells) and C_{117} (cyan cell), to which respectively three and one go. Thus, we get the matrix elements $P_{57,23} = 0.75$, $P_{57,117} = 0.25$, and $P_{57,j} = 0$ for all other j.

The *transistion matrix* is the Ulam matrix $\boldsymbol{P}$ with general element $P_{i,j}$. (Our Ulam matrix approximation of transfer operator $\boldsymbol{P}$ contains transition probabilities, and therefore can be considered a probability transition matrix. However, unlike in more standard implementations, it is not square, and is non-global, since we ignore the impact of particles outside the original seeding.) A schematic example of such a calculation is shown in Figure S2.

4. The matrix $\boldsymbol{P}$ approximates the transfer operator in the following sense: given any mass distribution across the cells $\{B_i\}$ encoded in an m-dimensional row vector $\boldsymbol{M}$, its eventual distribution at the final time is obtained by the matrix multiplication $\boldsymbol{MP}$. Note that the "operation" of $\boldsymbol{P}$ on $\boldsymbol{M}$ needs to be written in this order, and the n-dimensional row vector $\boldsymbol{MP}$ represents the distribution in the final cells $\{C_j\}$. (This is because here we represent the mass distribution $\boldsymbol{M}$ as a *row*-vector, and $\boldsymbol{P}$ is *row*-stochastic (the row sum $\sum_{j=1}^{m} P_{i,j} = 1$ for all i since a particle starting in cell B_i has to go *somewhere*). If $\boldsymbol{M}$ were a *column*-vector and $\boldsymbol{P}$ were constructed to be *column*-stochastic instead, the operation of $\boldsymbol{P}$ on $\boldsymbol{M}$ would need to be expressed by the matrix multiplication $\boldsymbol{PM}$.)
5. Mass is not uniformly distributed across an isentropic surface, and hence interpreting the transfer operator requires some care. We incorporate this mass variation in terms of pressure data as done in [8], and provide below the rationale for the appropriate weighting. Since being on an isentropic surface corresponds to adiabatic conditions, the pressure and volume obey

$$(\text{Pressure})\ (\text{Volume})^{\gamma} = \text{constant}\,,$$

where γ is the ratio of the heat capacity at constant pressure to that at constant volume. In atmospheric air which is mostly diatomic, $\gamma = 7/5$. This implies that

$$(\text{Pressure})^{1/\gamma} \, \frac{(\text{Mass})}{(\text{Density})} = \text{constant}\,,$$

and so for a chosen constant mass of air, we have that

$$(\text{Pressure})^{1/\gamma} \propto (\text{Density})\ .$$

Given that pressure measurements are available, the mass of air within a cell B_i is therefore proportional to

$$[\text{Density in}\,(B_i)]\ [\text{Area of}\,(B_i)] \propto [\text{Pressure in}\,(B_i)]^{1/\gamma}\ [\text{Area of}\,(B_i)]\ .$$

We define this quantity, the "mass weighting", as

$$\mu_i := [\text{Pressure in}\,(B_i)]^{1/\gamma}\ [\text{Area of}\,(B_i)]\ . \tag{3}$$

As in [11], we form the $m \times m$ diagonal matrix $\boldsymbol{\mu}$ by arranging the μ_i, $i = 1, \ldots, m$ along the diagonal. (The terms along the diagonal of $\boldsymbol{\mu}$ are positive, and therefore we can form powers of the matrix $\boldsymbol{\mu}$ by simply taking the appropriate power of each of the diagonal elements. Thus, the ith diagonal term of the diagonal matrix $\boldsymbol{\mu}^\alpha$, for any $\alpha \in \mathbb{R}$, would be μ_i^α. In particular, this works for determining the inverse $\boldsymbol{\mu}^{-1}$, as well as powers such as $\boldsymbol{\mu}^{1/2}$ or $\boldsymbol{\mu}^{-1/2}$ which we will need later. These are all $m \times m$ diagonal matrices.)

6. We can use the pressure measurements and areas in the final-time cells C_j, and similarly form a mass weighting matrix $\boldsymbol{\nu}$. However, an alternative is to observe that since the initial mass across all the cells $\{B_i\}$ is assumed to have been distributed to the final cells $\{C_j\}$, and $\boldsymbol{P}$ represents its transfer, we have the relationship

$$\left[\,\nu_1\ \ldots\ \nu_n\,\right] = \left[\,\mu_1\ \ldots\ \mu_m\,\right]\,\boldsymbol{P}\,. \tag{4}$$

That is, the final mass can be obtained by pushing forward the initial mass according to the probabilities of transition. In practice, this equality will only be approximately met, but to ensure that probabilities are respected within our numerical scheme, we compute the ν_j using (4) and prepare the $n \times n$ diagonal matrix $\boldsymbol{\nu}$ using these values rather than by using the pressure at the final time.

7. As defined, the transfer operator operates on *mass* distributions. Suppose we consider *parcel* distributions, that is, choosing *numbers* of "fluid parcels" in each cell. Let $\boldsymbol{u}$ be a parcel distribution in the initial $\{B_i\}$ cells, which means that the component u_i corresponds to the number of fluid parcels in the cell B_i for each $i = 1, 2, \ldots, m$. This would correspond to a mass in cell

B_i of $M_i = u_i \mu_i$, and hence we can identify the mass distribution at the initial time in terms of any given parcel distribution $\boldsymbol{u}$ by the m-dimensional row vector

$$\boldsymbol{M} = \boldsymbol{u}\,\boldsymbol{\mu}\,,$$

where $\boldsymbol{\mu}$ is the $m \times m$ diagonal matrix defined previously. Similarly, if $\boldsymbol{v}$ is a parcel distribution at the final time, i.e., with respect to the cells $\{C_j\}$, the corresponding mass distribution at the final time is given by the n-dimensional vector

$$\boldsymbol{M}' = \boldsymbol{v}\,\boldsymbol{\nu}\,,$$

where $\boldsymbol{\nu}$ is the $n \times n$ diagonal matrix encoding the mass weighting at the final time.

8. The crux of our problem is: how can we find the parcel distributions $\boldsymbol{u}$ and $\boldsymbol{v}$ at the initial and final times, corresponding to the "greatest coherence" with respect to the flow over that time period? These will respectively represent our Pre-Convective Modes and Convective Modes. To make sense of this intuitive question more concretely, we observe that given an initial parcel distribution $\boldsymbol{u}$, it would correspond to a mass distribution $\boldsymbol{u\mu}$, and consequently will get pushed by the flow to a mass distribution $\boldsymbol{u\mu P}$ at the final time. On the other hand, the mass distribution at the final time corresponding to a parcel distribution $\boldsymbol{v}$ at that time would be $\boldsymbol{v\nu}$. Hence, we would like to find the pair $\boldsymbol{u}$ and $\boldsymbol{v}$ which satisfy the approximate equality

$$\boldsymbol{u\mu P} \approx \boldsymbol{v\nu}\,. \tag{5}$$

9. In seeking solutions to (5), we need to normalize $\boldsymbol{u}$ and $\boldsymbol{v}$ appropriately. Since whatever fixed mass of air we have at the initial time must get transported to the final time, we need to think of $\boldsymbol{u}$ and $\boldsymbol{v}$ corresponding to the same mass. This can be accomplished by normalizing according to

$$\sum_{i=1}^{m} u_i^2 \mu_1 = 1 = \sum_{j=1}^{n} v_j^2 \nu_j\,. \tag{6}$$

The subtle interpretation here is that *the square of each term of $\boldsymbol{u}$ and $\boldsymbol{v}$ correspond to parcel distributions.* (The more direct interpretation without the square would require the normalization $\sum_{i=1}^{m} |u_i|\,\mu_i = 1$, which turns out to not give obvious mathematical solutions. We have effectively relaxed an ℓ^1-problem to an ℓ^2-problem, much like the issue of finding the trendline can be explicitly solved using least *square* as opposed to least *absolute-value* distances.) This enables $\boldsymbol{u}$ and $\boldsymbol{v}$ to possess both positive and negative terms to allow greater discernment within the distributions, while respecting the necessity for a parcel distribution to be non-negative.

10. The approximate equality (5) can be *exactly* satisfied if choosing $\boldsymbol{u}$ such that all its components are identical (i.e., a uniform mass distribution), and similarly for $\boldsymbol{v}$. This classical result from probability theory is because the full collection of parcels in $\sum_{i=1}^{m} B_i$ should get mapped to $\sum_{j=1}^{n} C_j$. We

exclude this uninformative solution, and seek other solutions which satisfy the approximate equality as closely as possible. In general, the velocities are only available on a grid, spatial interpolation and time-stepping are necessary in the numerical solution to the flow equation (1) resulting in *numerical diffusion*, and the presence of subgrid effects implies that there is effective diffusion on evolving fluid parcels. (Accounting for subgrid effects is sometimes done using the idea of "stochastic parameterization", which incorporates these effects in terms of a diffusion at the macroscopic scale [24, 25].) Taken together this means that there is "loss" when applying $\boldsymbol{P}$ to the initial mass distribution $\boldsymbol{u\mu}$ and in general, the left-hand side of (5) will not exceed the right-hand side. We therefore seek $\boldsymbol{u}$s and $\boldsymbol{v}$s that obey

$$\boldsymbol{u\mu P} = s\,\boldsymbol{v\nu} \quad 0 < s < 1\,. \tag{7}$$

This seeks vectors $\boldsymbol{u\mu P}$ and $\boldsymbol{v\nu}$ that are proportional to each other, and hence result in the same spatial structure when viewed as a colormap on the cells $\{C_j\}$. This would imply that a particular spatial distribution is a "coherent mode" for the flow. The closer s is to 1, the "better the coherence" between the pushed-forward initial mass distribution, and a legitimate distribution of that total mass at the final time. If the initial distribution was highly variable, i.e., had large gradients or filamented spatial structures, it would be much more susceptible to diffusion, and thereby less likely to be correctly representible at the final time by distributing the identical mass among the $\{C_j\}$ cells. In such cases, (7) may only be achieved for a small s.

11. As expressed, finding solutions $\boldsymbol{u}$, $\boldsymbol{v}$ and s to (7) subject to the normalizing conditions (6) is difficult. We therefore apply the transformations

$$\boldsymbol{u} = \hat{\boldsymbol{u}}\boldsymbol{\mu}^{-1/2} \quad , \quad \boldsymbol{v} = \hat{\boldsymbol{v}}\boldsymbol{\nu}^{-1/2} \quad \text{and} \quad \boldsymbol{P} = \boldsymbol{\mu}^{-1/2}\hat{\boldsymbol{P}}\boldsymbol{\nu}^{1/2} \tag{8}$$

in order to defined "hatted" vectors and operators. (This is effectively the approach suggested in [11], but we have motivated it quite differently, avoiding the concepts of weighted inner-product spaces and instead considering physical considerations such as total mass conservation, and $\boldsymbol{P}$ being a mass transport operator.) This results in

$$\left(\hat{\boldsymbol{u}}\boldsymbol{\mu}^{-1/2}\right)\boldsymbol{\mu}\left(\boldsymbol{\mu}^{-1/2}\hat{\boldsymbol{P}}\boldsymbol{\nu}^{1/2}\right) = s\,\left(\hat{\boldsymbol{v}}\boldsymbol{\nu}^{-1/2}\right)\boldsymbol{\nu}.$$

Simplifying, and multiplying on the right by $\boldsymbol{\nu}^{-1/2}$ reduces the equation to

$$\hat{\boldsymbol{u}}\,\hat{\boldsymbol{P}} = s\,\hat{\boldsymbol{v}}\,, \tag{9}$$

where by virtue of (8),

$$\hat{\boldsymbol{P}} = \boldsymbol{\mu}^{1/2}\boldsymbol{P}\boldsymbol{\nu}^{-1/2}\,. \tag{10}$$

12. The *Singular Value Decomposition (SVD)* for an $m \times n$ matrix $\hat{\boldsymbol{P}}$ is built in to many software platforms, and decomposes the operator $\hat{\boldsymbol{P}}$ as the product

$$\hat{\boldsymbol{P}} = \hat{\boldsymbol{U}}\,\boldsymbol{S}\,\hat{\boldsymbol{V}}^{\top}\,, \tag{11}$$

where $\hat{\boldsymbol{U}}$ is a unitary $m \times m$ matrix, $\hat{\boldsymbol{V}}$ is a unitary $n \times n$ matrix, and $\boldsymbol{S}$ is a rectangular diagonal matrix of dimensions $m \times n$ which has zero elements outside the diagonal. (A square matrix is unitary if its transpose is equal to its inverse.) In this representation, and with standard software implementations,

- The diagonal elements of $\boldsymbol{S}$ are the singular values of $\hat{\boldsymbol{P}}$, and will be returned by standard software as being non-negative, and arranged in descending order. There will be $\min\{m, n\}$ of these diagonal elements, because $\boldsymbol{S}$ is an $m \times n$ rectangular diagonal matrix.
- The ith column of $\hat{\boldsymbol{U}}$ is the left singular vector corresponding to the singular value $S_{i,i}$. It will be normalized such that the sum of the squares of its components is 1. (The ℓ^2-norm of a vector $\boldsymbol{w}$, denoted by $\|\boldsymbol{w}\|$, is the square root of the sum of the squares of the components of $\boldsymbol{w}$; this is equivalent to the vector's length.)
- The ith column of $\hat{\boldsymbol{V}}$ is the right singular vector corresponding to the singular value $S_{i,i}$. It will also be normalized such that the sum of the squares of its components is equal to 1.

By multiplying (11) on the left by $\hat{\boldsymbol{U}}^{-1} = \hat{\boldsymbol{U}}^{\top}$, the alternative representation of the SVD

$$\hat{\boldsymbol{U}}^{\top}\hat{\boldsymbol{P}} = \boldsymbol{S}\,\hat{\boldsymbol{V}}^{\top}\,.$$

can be obtained. The left and right singular vectors are now respectively the row vectors in $\hat{\boldsymbol{U}}^{\top}$ and $\hat{\boldsymbol{V}}^{\top}$. Observing that the ith row of this expression is therefore exactly in the form (9), we see that our required $\hat{\boldsymbol{u}}$, $\hat{\boldsymbol{v}}$ and s can be read off immediately from the SVD.

13. We know that and first left and right singular vectors (the first columns of each of $\hat{\boldsymbol{U}}$ and $\hat{\boldsymbol{V}}$) have components $\mu_i^{1/2}$ $(i = 1, 2, \ldots, m)$ and $\nu_j^{1/2}$ $(j = 1, 2, \ldots, n)$ respectively (the vectors $\boldsymbol{u}$ and $\boldsymbol{v}$ corresponding to the singular value 1 consist of uniform parcel distributions, so can be represented as having 1s as each component. However, by (8), this implies that the corresponding $\hat{\boldsymbol{u}}$ and $\hat{\boldsymbol{v}}$ are these particular forms of the weighting factors, as observed in [8, 11]), and that the corresponding singular value of $\hat{\boldsymbol{P}}$ is $S_{1,1} = 1$. (By construction we have ensured $\|\boldsymbol{x}\hat{\boldsymbol{P}}\|$ is maximised by $\boldsymbol{x} = \hat{\boldsymbol{u}}_0$, the first row of $\hat{\boldsymbol{U}}^{\top}$, where $\|\boldsymbol{x}\| = 1$. In which case $\hat{\boldsymbol{u}}_0\hat{\boldsymbol{P}} = [\mu_1^{1/2}, \ldots, \mu_m^{1/2}]\hat{\boldsymbol{P}} = [\nu_1^{1/2}, \ldots, \nu_n^{1/2}] = (S_{1,1})\hat{\boldsymbol{v}}_0$ and so $S_{1,1} = 1$ for $\hat{\boldsymbol{v}}_0$, the first row of $\hat{\boldsymbol{V}}^{\top}$.) The corresponding parcel distributions $\boldsymbol{u}$ and $\boldsymbol{v}$ are then uniform because of (8), and hence cannot tell us about the most coherent *spatial distributions*, which is what we seek. Thus, we need the "next modes". Given that the closer s in (9) is to 1, the more coherent the

relevant distributions are, we can therefore construct the following as our *most coherent modes.*

- The diagonal elements of $\boldsymbol{S}$ are identified as the ***coherence***, that is, the coherence of the kth mode is the relevant singular value

$$\mathrm{coh}_k := s_k = S_{k+1,k+1} \,, \tag{12}$$

  where standard software would have these arranged in descending order.
- The column vectors of $\hat{\boldsymbol{U}}$ are the left singular vectors of $\hat{\boldsymbol{P}}$, ordered according to the ordering of the singular values in $\boldsymbol{S}$. Ignoring the first column, we extract the next left singular vectors according to

$$\hat{\boldsymbol{u}}_k = (k+1)^{\mathrm{st}} \text{ column of } \hat{\boldsymbol{U}}\,, \text{ converted to a row vector}\,.$$

- Similarly, we can extract the relevant right singular vectors by

$$\hat{\boldsymbol{v}}_k = (k+1)^{\mathrm{st}} \text{ column of } \hat{\boldsymbol{V}}\,, \text{ converted to a row vector}\,.$$

- For each k, the pair of vectors $\boldsymbol{u} = \hat{\boldsymbol{u}}_k$ and $\boldsymbol{v} = \hat{\boldsymbol{v}}_k$ along with the singular value $s = s_k$ solves the problem (9). Given the number of diagonal elements of $\boldsymbol{S}$, and the fact that we exclude the first element, we know that k will take values in

$$k \in \{1, 2, 3, \ldots, \min\{m, n\} - 1\}$$

  and hence this process gives us $\min\{m, n\} - 1$ (one less than the smaller of m or n) different Convective Modes. We do not need to compute the singular values/vectors for all k, but only need the higher ones (say up to the 14th) because they correspond to highly coherent modes.

14. We can transform these results from the "hatted" variables to the original ones, by reversing the transformations in (8). The left singular vectors $\hat{\boldsymbol{u}}_k$ are

$$\mathrm{PCM}_k := \boldsymbol{u}_k = \hat{\boldsymbol{u}}_k \, \boldsymbol{\mu}^{-1/2} \,, \tag{13}$$

which we define to be ***Pre-Convective Modes (PCMs)***, each representing a coherent parcel distribution in the m cells $\{B_i\}$ at the initial time. The corresponding ***Convective Modes (CMs)***, demonstrating parcel distribution in the n cells $\{C_j\}$ at the final time, are defined by

$$\mathrm{CM}_k := \boldsymbol{v}_k = \hat{\boldsymbol{v}}_k \, \boldsymbol{\nu}^{-1/2} \,, \tag{14}$$

where the $\hat{\boldsymbol{v}}_k$s are the right singular vectors of $\hat{\boldsymbol{P}}$. Each k value has a pair $(\mathrm{PCM}_k, \mathrm{CM}_k)$, associated with a coherence value $s_k = \mathrm{coh}_k$. In view of (7) and the transformations (8), we see that for each k,

$$(\mathrm{PCM}_k \boldsymbol{\mu}) \, \boldsymbol{P} = \mathrm{coh}_k \, (\mathrm{CM}_k \boldsymbol{\nu}) \,, \tag{15}$$

which indicates the solutions, indexed by k, to the originally stated question of determining parcel distribution pairs in the initial and final time which are "most coherent." Specifically, the initial mass distribution $\mathrm{PCM}_k\boldsymbol{\mu}$ *is pushed forward by the transfer operator* $\boldsymbol{P}$ *to* coh_k *times the vector* $\mathrm{CM}_k\boldsymbol{\nu}$*, with modes associated with smaller values of* k *being more coherent.*

15. Since singular vectors are only defined up to a scalar multiple, most standard software will automatically normalize the columns of $\hat{\boldsymbol{U}}$ and $\hat{\boldsymbol{V}}$ such that their ℓ^2-norm in $\mathbb{R}^m$ and $\mathbb{R}^n$ respectively is unity. That is, for all $k = 1, 2, \dots, m-1$, each vector will have "length 1 ", i.e.,

$$\|\hat{\boldsymbol{u}}_k\| = 1 \quad \text{and} \quad \|\hat{\boldsymbol{v}}_k\| = 1\,.$$

However, from the PCM definition (13) we note that the ith component satisfies

$$\hat{\boldsymbol{u}}_{k,i} = \left[\mathrm{PCM}_k\,\boldsymbol{\mu}^{1/2}\right]_i = \mathrm{PCM}_{k,i}\,\mu_i^{1/2}$$

where the i-subscript pertains to the ith component of the relevant vector. Hence,

$$1 = \|\hat{\boldsymbol{u}}_k\|^2 = \sum_{i=1}^{m} \hat{\boldsymbol{u}}_{k,i}^2 = \sum_{i=1}^{m} \mathrm{PCM}_{k,i}^2\,\mu_i\,. \tag{16}$$

consistent with our physical interpretation (6) that *the square of a Pre-Convective Mode represents a distribution of parcels chosen so that the associated total mass of the parcels is normalized to* 1. Similarly,

$$1 = \|\hat{\boldsymbol{v}}_k\|^2 = \sum_{j=1}^{n} \hat{\boldsymbol{v}}_{k,j}^2 = \sum_{j=1}^{n} \mathrm{CM}_{k,j}^2\,\nu_j\,, \tag{17}$$

indicating that *the square of a Convective Mode is associated with a distribution of parcels at the final time such that the associated total mass of the parcels is* 1.

16. The presence of both positive and negative terms in the PCM and CM vectors allows for a more detailed understanding of the spatial structures. Consider any PCM_k and its corresponding CM_k, and separate space into each's positive and negative sets, i.e.,

$$\mathrm{PCM}_k^+ := \bigcup_{i=1}^{m}\{B_i\,:\,\mathrm{PCM}_{k,i}\geq 0\}\,,\; \mathrm{PCM}_k^- := \bigcup_{i=1}^{m}\{B_i\,:\,\mathrm{PCM}_{k,i}< 0\}\,,$$
$$\mathrm{CM}_k^+ := \bigcup_{j=1}^{n}\{C_j\,:\,\mathrm{CM}_{k,j}\geq 0\}\,,\quad \mathrm{CM}_k^- := \bigcup_{j=1}^{n}\{C_j\,:\,\mathrm{CM}_{k,j}< 0\}\,,$$

where the second subscript means the component of the vector. If the partitions into cells $\{B_i\}$ and $\{C_j\}$ at the initial and final times was sufficiently fine, then the mass from each cell will move exactly to one cell under the operation of $\boldsymbol{P}$ because there was no uncertainty, and diffusion would be

minimal. In this idealized situation, $\boldsymbol{P}$ would have exactly one 1 in each column, with all other terms being 0s. For example, suppose $P_{i,j} = 1$ but $P_{l,j} = 0$ for all $l \neq i$. Then, taking the jth component of (15) we see that

$$\mathrm{PCM}_{k,i}\,\mu_i = \mathrm{coh}_k\,\mathrm{CM}_{k,j}\,\nu_j\,.$$

Given the positivity of μ_i, ν_j and coh_k, this implies that PCM_k^+ must map to CM_k^+, and that PCM_k^- must map to CM_k^-. Hence, in this idealized situation we expect the positive and negative parts of the singular vectors to remain separated, and move as distinct coherent entities. In realistic situations of finite-sized cells, this claim is only approximately met, but has motivated the separation of the dominant mode (P)CM_1 along its zero contour into two "almost coherent sets" with respect to the flow from the initial to final times [11, 17]. (This idea has been extended in several ways to partition space into more than one coherent set [26, 27].)

Here, we do not seek a partition of space, but rather observe that this almost separation implies that we can *separately view the square of the* (P)CM_k *in each of the positive and negative sets as distributions of air parcels associated with a coherence level* coh_k.

17. All the above results are based on the transfer operator $\boldsymbol{P}$ being a *row-stochastic* one, with $P_{i,j}$ representing the transition from cell B_i to cell C_j. The analysis goes through if $\boldsymbol{P}$ were instead represented as a *column-stochastic* one, with $P_{j,i}$ now representing the transition from B_i to C_j. This alternative formulation is also common in the literature. In this case, we need to use *column vectors* (say $\boldsymbol{w}$) for all our relevant quantities, and have the operation of $\boldsymbol{P}$ (and similar operators) on $\boldsymbol{w}$ be in the opposite order, i.e., $\boldsymbol{Pw}$. All equations presented above would need to be rewritten in this alternative formulation. However, the end result is the same: PCM_k and CM_k (now as column vectors) represent the relevant (Pre-)Convective Modes, with the coherence s_k, all computed from the SVD of $\hat{P}$. (However, one needs to be consistent with the interpretation of left and right singular vectors in this case.)
18. In the above, the initial time t was any value. We can allow it to be general, and do the calculations for varying values of t. This is associated with the flow from a general time t due to the velocities over the time-window $[t, t+\tau]$, with t varying, and is the *rolling window* (or sliding window) approach suggested by [28, 29].

Algorithm 1 summarises the procedure described throughout Section 1.2. This algorithm highlights the key calculations needed to construct a chosen number (N) of (Pre-)Convective Mode vectors (PCM and CM) as well as their associated coherence (coh) and evolved cell collections ($\mathcal{C}$).

Algorithm 1 The construction of coherent Convective Modes

Require: Chosen number of modes N, relevant vector field **vel** and corresponding partition of the southern hemisphere $\{B_i\}_{i=1}^{d}$ with associated cell-center pressure values (Pres) and cell areas (Area) as well as timespan extremities t_1, t_F.

Ensure: Ordered collections $\{\mathrm{coh}_{k,t}\}$, $\{\mathrm{PCM}_{k,t}\}$, $\{\mathrm{CM}_{k,t}\}$, $\{\mathcal{C}_t\}$

function ConstructConvectiveModes
    Define initial collection of cells $\{B_i\}_{i=1}^{m}$
    for $t \leftarrow t_1$ to t_F **do**
        Integrate **vel**, determine $\mathcal{C} = \{C_j\}_{j=1}^{n}$ ▷ Equation (1)
        Calculate $\boldsymbol{P}$ ▷ Equation (2)
        Set $\mu_i = \mathrm{Pres}_i^{5/7} \times \mathrm{Area}\,(B_i)$ ▷ Equation (3)
        Define $\left[\, \nu_1 \;\ldots\; \nu_n \,\right] = \left[\, \mu_1 \;\ldots\; \mu_m \,\right] \boldsymbol{P}$ ▷ Equation (4)
        Build diagonal matrices $\boldsymbol{\mu}$, $\boldsymbol{\nu}$ such that $\mu_{i,i} = \mu_i$ and $\nu_{j,j} = \nu_j$
        Construct $\hat{\boldsymbol{P}} = \boldsymbol{\mu}^{1/2}\, \boldsymbol{P}\, \boldsymbol{\nu}^{-1/2}$ ▷ Equation (10)
        $[\,\hat{\boldsymbol{U}},\, \boldsymbol{S},\, \hat{\boldsymbol{V}}\,] = \mathrm{svds}\,(\hat{\boldsymbol{P}},\, N+1)$ ▷ Equation (11)
        for $k \leftarrow 1$ to N **do**
            $\mathrm{coh}_{k,t} = S_{k+1,k+1}$ ▷ Equation (12)
            $\mathrm{PCM}_k = \hat{\boldsymbol{u}}_k\, \boldsymbol{\mu}^{-1/2}$ ▷ Equation (13)
            $\mathrm{CM}_k = \hat{\boldsymbol{v}}_k\, \boldsymbol{\nu}^{-1/2}$ ▷ Equation (14)
            ▷ $\hat{\boldsymbol{u}}_k$ is the $(k+1)$th row of $\hat{\boldsymbol{U}}^\top$ and $\hat{\boldsymbol{v}}_k$ the $(k+1)$th row of $\hat{\boldsymbol{V}}^\top$
        end for
    end for
    return $\{\mathrm{coh}_{k,t}\}_{1\le k\le N, t_1\le t\le t_F}$, $\{\mathrm{PCM}_{k,t}\}_{1\le k\le N, t_1\le t\le t_F}$, $\{\boldsymbol{\mu}_t\}_{t_1\le t\le t_F}$, $\{\mathrm{CM}_{k,t}\}_{1\le k\le N, t_1\le t\le t_F}$, $\{\mathcal{C}_t\}_{t_1\le t\le t_F}$
end function

1.3 Tracking and lifespan identification

For each of the years 1999, 2002, 2019 and 2022, our analysis covers the time frame from $t_1 =$ August 1, 0000 UTC to $t_F =$ December 1, 0000 UTC with time increments of $\Delta t = 1$ hour. Algorithm 2 describes the method used to pair modes through time whilst Algorithm 3 illustrates how the lifespan of the Special Convective Mode (ΔSCM) is determined. Our analysis considers the leading 15 singular vectors and so we take $N = 14$ in Algorithm 2.

Algorithm 2 aims at tracking the evolution of Convective Modes through time, according to a minimization in the amount of mass carried by the predominant signed cluster. That is, we identify the cluster that contains the maximum amount of mass at a given time from within either the positive or negative components only. We then compare this to the mass contained by modes in this same area at the next time step. For the k-means clustering we choose to identify $c = 3$ clusters with an initial position of the 25th, 50th and 75th percentile of the relevant vector. We set the MATLAB empty action option to singleton and utilise no more than 1000 iterations. The bin numbers corresponding to the indices of the xth clusters components are $\{\mathrm{Id}_x\} \subset \{B_i\}$. A wide tilde is used to indicate tracked objects with $\{\widetilde{\mathrm{sign}}_{k,t}\}$ recording the most appropriate sign for the kth tracked mode at time t. One notes that the second subscript now denotes the time dependent component of each set.

Algorithm 3 takes the tracked modes $\{\widetilde{\mathrm{PCM}}_{k,t}\}$ and associated coherence $\{\widetilde{\mathrm{coh}}_{k,t}\}$ returned by Algorithm 2 and determines the relevant path and lifespan of the special convective mode $\{\mathrm{SCM}_t\}$. In all four years, it is evident that the SPV is evolving coherently at time t_1 in the leading (P)CM where $n = 1$. This is taken as the initial condition for the tracking in Algorithm 3. The lifespan cut-off threshold *thresh* is taken to be twice the cost of transitioning from the minimising kth to lth position of dominance at the initial time $\widetilde{\mathrm{cost}}_{n,t_1}$ for the chosen tracked mode of initial position n (line 4, Algorithm 3).

Line 8 of Algorithm 3 defines the time window that signals anomalous change ΔSCM. This indicates the time window that follows the final time window within which Algorithm 3 identifies a coherently evolving structure in the Special Convective Mode SCM. One notes that $\{\mathrm{SCM}\}_{t_1 \leq t \leq t_f - \Delta t}$ is illustrated by a red line in the figure in the main document tracking the evolution of the coherence values for each year. This approach allows one to consider changes in the time dependent coherence of structures that exist over fixed time intervals. Whilst Algorithm 3 is concerned only with the lifespan of the SPV, this algorithm has the potential to also identify the existence of multiple, asynchronous lifespans.

Algorithm 2 Tracking modes through time

Require: Ordered collections $\{\mathrm{PCM}_{k,t}\}$, $\{\boldsymbol{\mu}_t\}$ from Algorithm 1 alongside chosen number of clusters c, modes N and times t_1, t_F.

Ensure: Tracked modes $\{\widetilde{\mathrm{PCM}}_{k,t}\}$, associated coherence $\{\widetilde{\mathrm{coh}}_{k,t}\}$, transition cost $\{\widetilde{\mathrm{cost}}_{k,t}\}$ and mode signs $\{\widetilde{\mathrm{sign}}_{k,t}\}$

function TrackModes($\{\mathrm{PCM}_{k,t}\}$, c, N, t_1, t_F)
  $\mathcal{N} \leftarrow \{1, \ldots, N\}$, $m \leftarrow \mathrm{length}\,(\mathrm{PCM}_{1,1})$
  $\{pos_{k,t_1}\} \leftarrow \mathcal{N}$, $\{\mathrm{sign}_{k,t_1}\} \in \{-1, 1\}$ ▷ Set *natural* initial positions
  for $t \leftarrow t_1$ to $t_F - \Delta t$ **do**
    for $k \leftarrow 1$ to $\mathcal{N}$ **do**
      for $i \leftarrow 1$ to m **do** ▷ Create signed partitions
        $\left(\mathrm{PCM}^{+}\right)_i \leftarrow \max\left(\left(\mathrm{PCM}_{k,t}\right)_i, 0\right)$
        $\left(\mathrm{PCM}^{*}\right)_i \leftarrow \max\left(0, -\left(\mathrm{PCM}_{k,t}\right)_i\right)$
      end for
      if $\sum_{i=1}^{m}\left(\mathrm{PCM}^{+}\right)_i^2 > \sum_{i=1}^{m}\left(\mathrm{PCM}^{*}\right)_i^2$ **then**
        $\mathrm{PCM}^{*} \leftarrow \mathrm{PCM}^{+}$ ▷ Identify partition of greatest interest
      end if
      $\{\mathrm{Id}_1, \ldots, \mathrm{Id}_c\} = \mathrm{kmeans}\,(\mathrm{PCM}^{*}, c)$
        ▷ Determine membership indices for c clusters of partition
      $\mathrm{maxMass} \leftarrow \arg\max_{1 \leq x \leq c} \sum_{i \in \mathrm{Id}_x} \left(\mathrm{PCM}^{*}\right)_i^2$
        ▷ Identify cluster of largest mass
      $\underline{\mathrm{PCM}} \leftarrow \left\{\left(\mathrm{PCM}_{k,t}\right)_i : i \in \mathrm{Id}_{\mathrm{maxMass}}, 0 \text{ otherwise}\right\}$
        ▷ Isolate relevant cluster using *mask* given by $\mathrm{Id}_{\mathrm{maxMass}}$
      for $l \leftarrow 1$ to $\mathcal{N}$ **do**
        $\overline{\mathrm{PCM}} \leftarrow \left\{\left(\mathrm{PCM}_{l,t+\Delta t}\right)_i : i \in \mathrm{Id}_{\mathrm{maxMass}}, 0 \text{ otherwise}\right\}$
          ▷ Isolate clustered region at following time-step
        $\mathbf{C}_{k,l} \leftarrow \left(\sum_{i=1}^{m}\left|\,(\underline{\mathrm{PCM}})_i^2(\mu_t)_{i,i} - (\overline{\mathrm{PCM}})_i^2(\mu_{t+\Delta t})_{i,i}\,\right|\right)^{1/2}$
          ▷ Cost of transition, *normalised* as per Equation (16)
      end for
    end for
    for $k \leftarrow 1$ to $\mathcal{N}$ **do**
      $[\mathrm{row}, \mathrm{col}] \leftarrow \arg\min_{i,j \in \mathcal{N}} \mathbf{C}_{i,j}$ ▷ Transition that minimises cost
      $\mathrm{pos}_{\mathrm{row},t+\Delta t} \leftarrow \mathrm{col}$
      $\mathrm{cost}_{\mathrm{row},\mathrm{col},t} \leftarrow \mathbf{C}_{\mathrm{row},\mathrm{col}}$
      $\mathrm{sign}_{\mathrm{row},t+\Delta t} \leftarrow \arg\min_{s \in \{+1,-1\}} \|\mathrm{sign}_{\mathrm{row},t}\mathrm{PCM}_{\mathrm{row},t} + s\, \mathrm{PCM}_{\mathrm{col},t+\Delta t}\|$
        ▷ Determine sign of future mode
      Set elements $\{\mathbf{C}_{\mathrm{row},j}\}_{j=1}^{N}$ and $\{\mathbf{C}_{i,\mathrm{col}}\}_{i=1}^{N}$ to ∞
    end for
  end for
  Use $\{\mathrm{pos}_{k,t}\}$ to collate time ordered tracked modes $\{\widetilde{\mathrm{PCM}}_{k,t}\}$ with associated tracked coherence $\{\widetilde{\mathrm{coh}}_{k,t}\}$, $\{\widetilde{\mathrm{cost}}_{k,t}\}$ and minimising $\{\widetilde{\mathrm{sign}}_{k,t}\}$
  return $\{\widetilde{\mathrm{PCM}}_{k,t}\}_{1 \leq k \leq N, t_1 \leq t \leq t_F}$, $\{\widetilde{\mathrm{coh}}_{k,t}\}_{1 \leq k \leq N, t_1 \leq t \leq t_F}$, $\{\widetilde{\mathrm{sign}}_{k,t}\}_{1 \leq k \leq N, t_1 \leq t \leq t_F}$, $\{\widetilde{\mathrm{cost}}_{k,t}\}_{1 \leq k \leq N, t_1 \leq t \leq t_F}$
end function

Algorithm 3 Isolating lifespan of Special Convective Mode

Require: $\{\widetilde{\mathrm{PCM}}_{n,t}\}$, coherence $\{\widetilde{\mathrm{coh}}_{n,t}\}$, and $\{\widetilde{\mathrm{cost}}_{n,t}\}$ from Algorithm 2 for chosen mode of initial position $n \in \mathcal{N}$, time extremities t_1, t_F and thresh.
Ensure: Special Convective Mode $\{\mathrm{SCM}_t\}$ and associated path, as defined by the relevant coherence values

```
function LIFESPAN({P̃CM_{n,t}}, {c̃oh_{n,t}}, {c̃ost_{n,t}}, thresh)
    SCM_{t_1} ← P̃CM_{n,t_1}                                   ▷ Set initial conditions
    for t ← t_1 + Δt to t_F do
        if (c̃ost_{n,t−Δt} / c̃ost_{n,t_1}) ≤ thresh then
            SCM_t ← P̃CM_{n,t} with associated coherence
        else
            Define final lifespan instance t_f ← t − Δt
            ΔSCM ← [t_f, t_f + τ]                           ▷ Anomalous change window
            return {SCM_t}_{t_1 ≤ t ≤ t_f − Δt}, associated coherence and ΔSCM
        end if
    end for
    return {SCM_t}_{t_1 ≤ t ≤ t_F}, associated coherence and ΔSCM undefined
end function
```

1.4 Visualisation

Included in the supplementary material for this research are animations that illustrate our results for each year at 12 hourly time intervals. These animations consist of the following four movies:

- Movie S1: Evolving Convective Modes and Atmospheric Variables, 1999.
- Movie S2: Evolving Convective Modes and Atmospheric Variables, 2002.
- Movie S3: Evolving Convective Modes and Atmospheric Variables, 2019.
- Movie S4: Evolving Convective Modes and Atmospheric Variables, 2022.

As is the case throughout this paper, these animations compare the evolution of Convective Modes with that of the instantaneous measurements of the physical quantities ozone, potential vorticity and temperature. The sign for each CM illustrated is chosen using the minimisation technique described on line 25, Algorithm 2 for the untracked modes with $\Delta t = 12$h. For consistency across time, all (Pre-)Convective modes and physical variables are plotted using a constant color axis for all longitude values south of $-22°$ in latitude.

References

[1] Hersbach, H., Bell, B., Berrisford, P., Hirahara, S., Horányi, A., Muñoz-Sabater, J., Nicolas, J., Peubey, C., Radu, R., Schepers, D., Simmons, A., Soci, C., Abdalla, S., Abellan, X., Balsamo, G., Bechtold, P., Biavati, G., Bidlot, J., Bonavita, M., Chiara, G., Dahlgren, P., Dee, D., Diamantakis, M., Dragani, R., Flemming, J., Forbes, R., Fuentes, M., Geer, A., Haimberger, L., Healy, S., Hogan, R.J., Hólm, E., Janisková, M., Keeley, S., Laloyaux, P., Lopez, P., Lupu, C., Radnoti, G., Rosnay, P., Rozum, I., Vamborg, F., Villaume, S., Thépaut, J.: The ERA5 global reanalysis. Quarterly journal of the Royal Meteorological Society **146**(730), 1999–2049 (2020)

[2] Saha, S., Moorthi, S., Pan, H.-L., Wu, X., Wang, J., Nadiga, S., Tripp, P., Kistler, R., Woollen, J., Behringer, D., Liu, H., Stokes, D., Grumbine, R., Gayno, G., Wang, J., Hou, Y.-T., Chuang, H.-Y., Juang, H.-M.H., Sela, J., Iredell, M., Treadon, R., Kleist, D., Van Delst, P., Keyser, D., Derber, J., Ek, M., Meng, J., Wei, H., Yang, R., Lord, S., van den Dool, H., Kumar, A., Wang, W., Long, C., Chelliah, M., Xue, Y., Huang, B., Schemm, J.-K., Ebisuzaki, W., Lin, R., Xie, P., Chen, M., Zhou, S., Higgins, W., Zou, C.-Z., Liu, Q., Chen, Y., Han, Y., Cucurull, L., Reynolds, R.W., Rutledge, G., Goldberg, M.: NCEP Climate Forecast System Reanalysis (CFSR) 6-hourly Products, January 1979 to December 2010. Research Data Archive at the National Center for Atmospheric Research, Computational and Information Systems Laboratory, Boulder CO (2010). https://doi.org/10.5065/D69K487J

[3] Saha, S., Moorthi, S., Wu, X., Wang, J., Nadiga, S., Tripp, P., Behringer,

D., Hou, Y.-T., Chuang, H.-y., Iredell, M., Ek, M., Meng, J., Yang, R., Mendez, M.P., van den Dool, H., Zhang, Q., Wang, W., Chen, M., Becker, E.: NCEP Climate Forecast System Version 2 (CFSv2) 6-hourly Products. Research Data Archive at the National Center for Atmospheric Research, Computational and Information Systems Laboratory, Boulder CO (2011). https://doi.org/10.5065/D61C1TXF

[4] Newman, P., Nash, E.: NASA Ozone Watch: Images, data, and information for atmospheric ozone. https://ozonewatch.gsfc.nasa.gov/meteorology/SH.html. Accessed: 3 May 2023 (2023)

[5] Balasuriya, S., Ouellette, N., Rypina, I.: Generalized Lagrangian coherent structures. Physica D: Nonlinear Phenomena **37**, 31–51 (2018)

[6] Hadjighasem, A., Farazmand, M., Balzevski, D., Froyland, G., Haller, G.: A critical comparison of Lagrangian methods for coherent structure detection. Chaos **27**, 053104 (2017)

[7] Peacock, T., Dabiri, J.: Introduction to Focus Issue: Lagrangian coherent structures. Chaos **20**, 017501 (2010)

[8] Santitissadeekorn, N., Froyland, G., Monahan, A.: Optimally coherent sets in geophysical flows: a transfer-operator approach to delimiting the stratospheric polar vortex. Physical Review. E, Statistical, nonlinear, and soft matter physics **82**(5 Pt 2), 056311–056311 (2010)

[9] Ndour, M., Padberg-Gehle, K., Rasmussen, M.: Spectral early-warning signals for sudden changes in time-dependent flow patterns. Fluids **6**(2) (2021). https://doi.org/10.3390/fluids6020049

[10] Huisinga, W., Schmidt, B.: In: Leimkuhler, B., Chipot, C., Elber, R., Laaksonen, A., Mark, A., Schlick, T., Schütte, C., Skeel, R. (eds.) Metastability and dominant eigenvalues of transfer operators, pp. 167–182. Springer, Berlin, Heidelberg (2006). https://doi.org/10.1007/3-540-31618-3_11. https://doi.org/10.1007/3-540-31618-3_11

[11] Froyland, G., Santitissadeekorn, N., Monahan, A.: Transport in time-dependent dynamical systems: finite-time coherent sets. Chaos **20**(4), 043116–104311610 (2010). https://doi.org/10.1063/1.3502450

[12] Xu, M., Paul, M.R.: Covariant Lyapunov vectors of chaotic Rayleigh-Bénard convection. Physical Review E **93**, 062208 (2016). https://doi.org/10.1103/PhysRevE.93.062208

[13] Klünker, A., Schneide, C., Froyland, G., Schumacher, J., Padberg-Gehle, K.: Set-oriented and finite-element study of coherent behavior in Rayleigh-Bénard convection. In: Junge, O., Schütze, O., Froyland,

G., Ober-Blöbaum, S., Padberg-Gehle, K. (eds.) Advances in Dynamics, Optimization and Computation, pp. 86–108. Springer, Cham (2020)

[14] Beckers, B., Beckers, P.: A general rule for disk and hemisphere partition into equal-area cells. Computational Geometry **45**(7), 275–283 (2012). https://doi.org/10.1016/j.comgeo.2012.01.011

[15] Ulam, S.M.: A Collection of Mathematical Problems. Interscience tracts in pure and applied mathematics; no. 8. Interscience Publishers, New York (1960)

[16] Raghunathan, M.S.: A proof of Oseledec's multiplicative ergodic theorem. Israel Journal of Mathematics **32**(4), 356–362 (1979)

[17] Dellnitz, M., Junge, O.: On the approximation of complicated dynamical behavior. SIAM Journal on Numerical Analysis **36**(2), 491–515 (1999) https://arxiv.org/abs/https://doi.org/10.1137/S0036142996313002. https://doi.org/10.1137/S0036142996313002

[18] Froyland, G.: Ulam's method for random interval maps. Nonlinearity **12**(4), 1029 (1999). https://doi.org/10.1088/0951-7715/12/4/318

[19] Blank, M., Keller, G.: Random perturbations of chaotic dynamical systems: stability of the spectrum. Nonlinearity **11**(5), 1351 (1998). https://doi.org/10.1088/0951-7715/11/5/010

[20] Li, T.: Finite approximation for the Frobenius-Perron operator. a solution to Ulam's conjecture. Journal of Approximation Theory **17**(2), 177–186 (1976)

[21] Froyland, G., Lloyd, S., Quas, A.: Coherent structures and isolated spectrum for Perron-Frobenius cocycles. Ergodic Theory and Dynamical Systems **30**(3), 729–756 (2010). https://doi.org/10.1017/S0143385709000339

[22] Dellnitz, M., Froyland, G., Junge, O.: The algorithms behind GAIO — set oriented numerical methods for dynamical systems. In: Fiedler, B. (ed.) Ergodic Theory, Analysis, and Efficient Simulation of Dynamical Systems, pp. 145–174. Springer, Berlin, Heidelberg (2001)

[23] Froyland, G.: An analytic framework for identifying finite-time coherent sets in time-dependent dynamical systems. Physica D: Nonlinear Phenomena **250**, 1–19 (2013). https://doi.org/10.1016/j.physd.2013.01.013

[24] Gottwald, G.A., Peters, K., Davies, L.: A data-driven method for the stochastic parametrisation of subgrid-scale tropical convective area fraction. Quarterly journal of the Royal Meteorological Society **142**(694),

349–359 (2016)

[25] Polzin, R., Müller, A., Rust, H., Névir, P., Koltai, P.: Direct Bayesian model reduction of smaller scale convective activity conditioned on large-scale dynamics. Nonlinear Processes in Geophysics **29**(1), 37–52 (2022). https://doi.org/10.5194/npg-29-37-2022

[26] Froyland, G., Lloyd, S., Santitissadeekorn, N.: Coherent sets for nonautonomous dynamical systems. Physica D: Nonlinear Phenomena **239**(16), 1527–1541 (2010)

[27] Froyland, G., Padberg-Gehle, K.: Almost-invariant and finite-time coherent sets: Directionality, duration, and diffusion. In: Bahsoun, W., Bose, C., Froyland, G. (eds.) Ergodic Theory, Open Dynamics, and Coherent Structures, pp. 171–216. Springer, New York, NY (2014)

[28] Blachut, C., González-Tokman, C.: A tale of two vortices: How numerical ergodic theory and transfer operators reveal fundamental changes to coherent structures in non-autonomous dynamical systems. Journal of Computational Dynamics **7**(2), 369 (2020). https://doi.org/10.3934/jcd.2020015

[29] Blachut, C., González-Tokman, C., Hernández-Dueñas, G.: A patch in time saves nine: Methods for the identification of localised dynamical behaviour and lifespans of coherent structures. Journal of Nonlinear Science **33**(4) (2023)
