# Supplementary material for: Convective modes reveal the incoherence of the Southern Polar Vortex
Source: Sci Rep. 2024 Jan 10;14:966. doi: 10.1038/s41598-023-50411-x (PMC10782018; doi:10.1038/s41598-023-50411-x)
Supplement: Supplementary file 6 — Supplementary Legends. [file 41598_2023_50411_MOESM6_ESM.docx]

Please find the following supplementary material included:

1. **Supplementary material document:** A detailed, yet intuitive description of how Convective Modes are constructed and why. The complex mathematics are explained intuitively so that non-experts can reconstruct and extend our methods to appropriate applications within their own fields. The document also includes an outline of the algorithms necessary to reproduce this work.
2. **Movie S1: Evolving Convective Modes and Atmospheric Variables, 1999:** An animation of the leading 3 Convective Modes for the time period August 15 1999 to December 1 1999. Convective Modes are presented alongside 3 important atmospheric variables (Ozone, Temperature, Potential Vorticity) for direct comparison. For brevity the modes at plotted at 12 hourly intervals.
3. **Movie S2: Evolving Convective Modes and Atmospheric Variables, 2002**: An animation of the leading 3 Convective Modes for the time period August 15 2002 to December 1 2002. Convective Modes are presented alongside 3 important atmospheric variables (Ozone, Temperature, Potential Vorticity) for direct comparison. For brevity the modes at plotted at 12 hourly intervals.
4. **Movie S3: Evolving Convective Modes and Atmospheric Variables, 2019:** An animation of the leading 3 Convective Modes for the time period August 15 2019 to December 1 2019. Convective Modes are presented alongside 3 important atmospheric variables (Ozone, Temperature, Potential Vorticity) for direct comparison. For brevity the modes at plotted at 12 hourly intervals.
5. **Movie S4: Evolving Convective Modes and Atmospheric Variables, 2022:** An animation of the leading 3 Convective Modes for the time period August 15 2022 to December 1 2022. Convective Modes are presented alongside 3 important atmospheric variables (Ozone, Temperature, Potential Vorticity) for direct comparison. For brevity the modes at plotted at 12 hourly intervals.
